# A Germline-Targeting Chimpanzee SIV Envelope Glycoprotein Elicits a New Class of V2-Apex Directed Cross-Neutralizing Antibodies

**DOI:** 10.1128/mbio.03370-22

**Published:** 2023-01-11

**Authors:** Frederic Bibollet-Ruche, Ronnie M. Russell, Wenge Ding, Weimin Liu, Yingying Li, Kshitij Wagh, Daniel Wrapp, Rumi Habib, Ashwin N. Skelly, Ryan S. Roark, Scott Sherrill-Mix, Shuyi Wang, Juliette Rando, Emily Lindemuth, Kendra Cruickshank, Younghoon Park, Rachel Baum, John W. Carey, Andrew Jesse Connell, Hui Li, Elena E. Giorgi, Ge S. Song, Shilei Ding, Andrés Finzi, Amanda Newman, Giovanna E. Hernandez, Emily Machiele, Derek W. Cain, Katayoun Mansouri, Mark G. Lewis, David C. Montefiori, Kevin J. Wiehe, S. Munir Alam, I-Ting Teng, Peter D. Kwong, Raiees Andrabi, Laurent Verkoczy, Dennis R. Burton, Bette T. Korber, Kevin O. Saunders, Barton F. Haynes, Robert J. Edwards, George M. Shaw, Beatrice H. Hahn

**Affiliations:** a Department of Medicine, University of Pennsylvania, Philadelphia, Pennsylvania, USA; b Theoretical Biology and Biophysics, Los Alamos National Laboratory, Los Alamos, New Mexico, USA; c Duke Human Vaccine Institute, Duke University School of Medicine, Durham, North Carolina, USA; d Department of Medicine, Duke University School of Medicine, Durham, North Carolina, USA; e Vaccine and Immunotherapy Center, The Wistar Institute, Philadelphia, Pennsylvania, USA; f Department of Immunology and Microbiology, The Scripps Research Institute, La Jolla, California, USA; g Centre de Recherche du CHUM, Montreal, Quebec, Canada; h Département de Microbiologie, Infectiologie et Immunologie, Université de Montréal, Montreal, Quebec, Canada; i Bioqual, Inc., Rockville, Maryland, USA; j Department of Surgery, Duke University School of Medicine, Durham, North Carolina, USA; k Vaccine Research Center, National Institute of Allergy and Infectious Diseases, National Institutes of Health, Bethesda, Maryland, USA; l San Diego Biomedical Research Institute, San Diego, California, USA; m Ragon Institute of MGH, Harvard and MIT, Cambridge, Massachusetts, USA; n Department of Microbiology, University of Pennsylvania, Philadelphia, Pennsylvania, USA; University of North Carolina at Chapel Hill

**Keywords:** SCIV, V2-apex, broadly neutralizing antibodies, chimpanzee, immunofocusing, germline-targeting, occluded-open trimer, human immunodeficiency virus, neutralizing antibodies, vaccines

## Abstract

HIV-1 and its SIV precursors share a broadly neutralizing antibody (bNAb) epitope in variable loop 2 (V2) at the envelope glycoprotein (Env) trimer apex. Here, we tested the immunogenicity of germ line-targeting versions of a chimpanzee SIV (SIVcpz) Env in human V2-apex bNAb heavy-chain precursor-expressing knock-in mice and as chimeric simian-chimpanzee immunodeficiency viruses (SCIVs) in rhesus macaques (RMs). Trimer immunization of knock-in mice induced V2-directed NAbs, indicating activation of V2-apex bNAb precursor-expressing mouse B cells. SCIV infection of RMs elicited high-titer viremia, potent autologous tier 2 neutralizing antibodies, and rapid sequence escape in the canonical V2-apex epitope. Six of seven animals also developed low-titer heterologous plasma breadth that mapped to the V2-apex. Antibody cloning from two of these animals identified multiple expanded lineages with long heavy chain third complementarity determining regions that cross-neutralized as many as 7 of 19 primary HIV-1 strains, but with low potency. Negative stain electron microscopy (NSEM) of members of the two most cross-reactive lineages confirmed V2 targeting but identified an angle of approach distinct from prototypical V2-apex bNAbs, with antibody binding either requiring or inducing an occluded-open trimer. Probing with conformation-sensitive, nonneutralizing antibodies revealed that SCIV-expressed, but not wild-type SIVcpz Envs, as well as a subset of primary HIV-1 Envs, preferentially adopted a more open trimeric state. These results reveal the existence of a cryptic V2 epitope that is exposed in occluded-open SIVcpz and HIV-1 Env trimers and elicits cross-neutralizing responses of limited breadth and potency.

## INTRODUCTION

Broadly neutralizing antibodies (bNAbs) represent a key defense against viruses and an important correlate of immune protection of antiviral vaccines (1). However, despite concerted efforts for nearly 3 decades, there are currently no HIV-1 immunogens that consistently elicit high titer bNAbs in outbred animals or humans (2 to 4). While humans have the capacity to develop bNAbs, breadth and potency usually appear only after years of HIV-1 infection and only in a subset of individuals (5 to 7). This is because most bNAbs have atypical features such as exceptionally long heavy chain complementarity determining region 3 (HCDR3) segments, high levels of somatic hypermutation, insertions or deletions in variable regions, and low precursor frequencies, all of which pose substantial barriers to traditional vaccine approaches (4, 8, 9). Nonetheless, the identification of bNAbs in HIV-1-infected humans, and more recently in simian-human immunodeficiency virus (SHIV)-infected rhesus macaques (RMs), has provided proof-of-principle that bNAb responses can be elicited and prompted studies to dissect the pathways of virus–antibody coevolution as a blueprint for vaccine design (10 to 16). It is widely believed that an effective vaccination strategy will need to stimulate rare precursor B cells of multiple bNAb lineages, which will then have to be affinity matured along desired pathways (4, 17, 18).

Env trimers display a large antigenic surface that can engage many different B cell receptors. Thus, immunogens that can focus B cell responses to specific epitopes may reduce unwanted off-target responses. We previously reported that certain strains of simian immunodeficiency viruses (SIVcpz and SIVgor) infecting chimpanzees (Pan troglodytes) and western gorillas (Gorilla gorilla) are exquisitely sensitive to neutralization by human variable loop 2 (V2)-directed bNAbs, but not bNAbs targeting V3 mannose patch, CD4 binding site (CD4bs), and interface epitopes (19). This antigenic conservation suggested that SIV-based immunogens might serve to immunofocus B cell responses to V2-apex epitopes shared by HIV-1. Indeed, a minimally modified near-native soluble SIVcpz Env trimer MT145.Q171K (abbreviated MT145K) was shown to bind inferred germ line precursors of several human V2-apex bNAbs and to stimulate one of these in a V2-apex bNAb germ line heavy chain (CH01^gH^) expressing knock-in mouse (20). Subsequent boosting with a cocktail of V2-apex sensitive HIV-1 Env trimers induced heterologous neutralizing antibodies in a subset of mice (20).

There are different strategies to examine the bNAb induction potential of HIV-1 Envs, one of which is in the context of simian-human immunodeficiency virus (SHIV) infection of RMs (12). SHIVs express HIV-1 Envs as functional trimers on the surface of infected cells and virions. Moreover, SHIVs replicate continuously over the course of the infection, resulting in an evolving viral quasispecies that can drive antibody somatic hypermutation and maturation (12, 21). Indeed, the patterns of envelope–antibody (Env-Ab) coevolution in SHIV-infected RMs are remarkably similar to those observed in HIV-1 infected humans, indicating similar mechanisms of epitope recognition and neutralizing antibody escape (12). Finally, SHIVs expressing V2-apex bNAb sensitive Envs commonly induce V2-directed neutralization breadth in RMs (12, 21). Thus, SHIV infection of RMs recapitulates V2-apex and other bNAb development in an animal model that closely approximates HIV-1-infected humans.

Immunofocusing is designed to reduce off-target responses by eliciting B cell recall responses to a shared epitope in otherwise antigenically different immunogens (22 to 26). Here, we examined the immunofocusing potential of Envs from diverse primate lentiviruses and found that several, including Envs from highly divergent SIVs infecting mustached (*Cercopithecus cephus*) and red-tailed (*Cercopithecus ascanius*) monkeys, were exquisitely sensitive to neutralization by mature human V2-apex bNAbs. However, these same Envs were resistant to neutralization by inferred ancestors of V2-apex bNAbs, even after the introduction of mutations previously shown to improve precursor binding (20). The exceptions were two V2-engineered SIVcpz Envs, CAM13.Q171K (CAM13K) and DP943.Q171K (DP943K), which, like the previously reported MT145K Env (20), were neutralized by the reverted unmutated ancestors (RUA) of PG9, PG16, and CH01. To examine their germ line-targeting potential, we selected one of these Envs (CAM13K) together with a further modified derivative (CAM13.K169R.K170R.Q171K or CAM13RRK) for immunogenicity studies in human V2-apex bNAb germ line-expressing knock-in mice and SCIV-infected RMs. Although the CAM13K SOSIP trimer expanded human V2-apex precursor-expressing mouse B cells and induced V2-directed neutralizing responses, neither SCIV.CAM13K nor SCIV.CAM13RRK induced prototypical V2-apex bNAbs in infected monkeys. Instead, SCIV infection elicited a novel V2-directed antibody specificity that cross-neutralized a number of tier 2 HIV-1 strains with low potency by recognizing a cryptic V2 epitope in occluded-open Env trimers (27, 28).

## RESULTS

### Antigenic conservation of the V2-apex among primate lentiviruses.

The apex of the closed prefusion HIV-1 Env trimer comprises the V1V2 loops of three gp120 protomers, which are positioned around its axis and form the top layer of the glycoprotein (29 to 32). Canonical V2-apex bNAbs access this protein surface through their long anionic HCDR3s that penetrate the glycan shield surrounding N-linked glycans at positions 160 (N160) and 156 (N156) and contact positively charged lysine residues in strand C (positions 168 to 171) of the V2 loop. Testing three human V2-apex bNAbs, we previously found that HIV-1 shares unexpected antigenic cross-reactivity in this epitope with certain SIVcpz and SIVgor strains (19). Here, we assembled 33 Envs from diverse primate lentiviruses and analyzed their sensitivity to a larger panel of V2-apex bNAbs, including the prototypic PG9, PG16, PGT145, PGDM1400, VRC26.25, and CH01 (29, 33 to 36) as well as BG1 and VRC38, which have shorter HCDR3 loops (37, 38).

**FIG 1:**
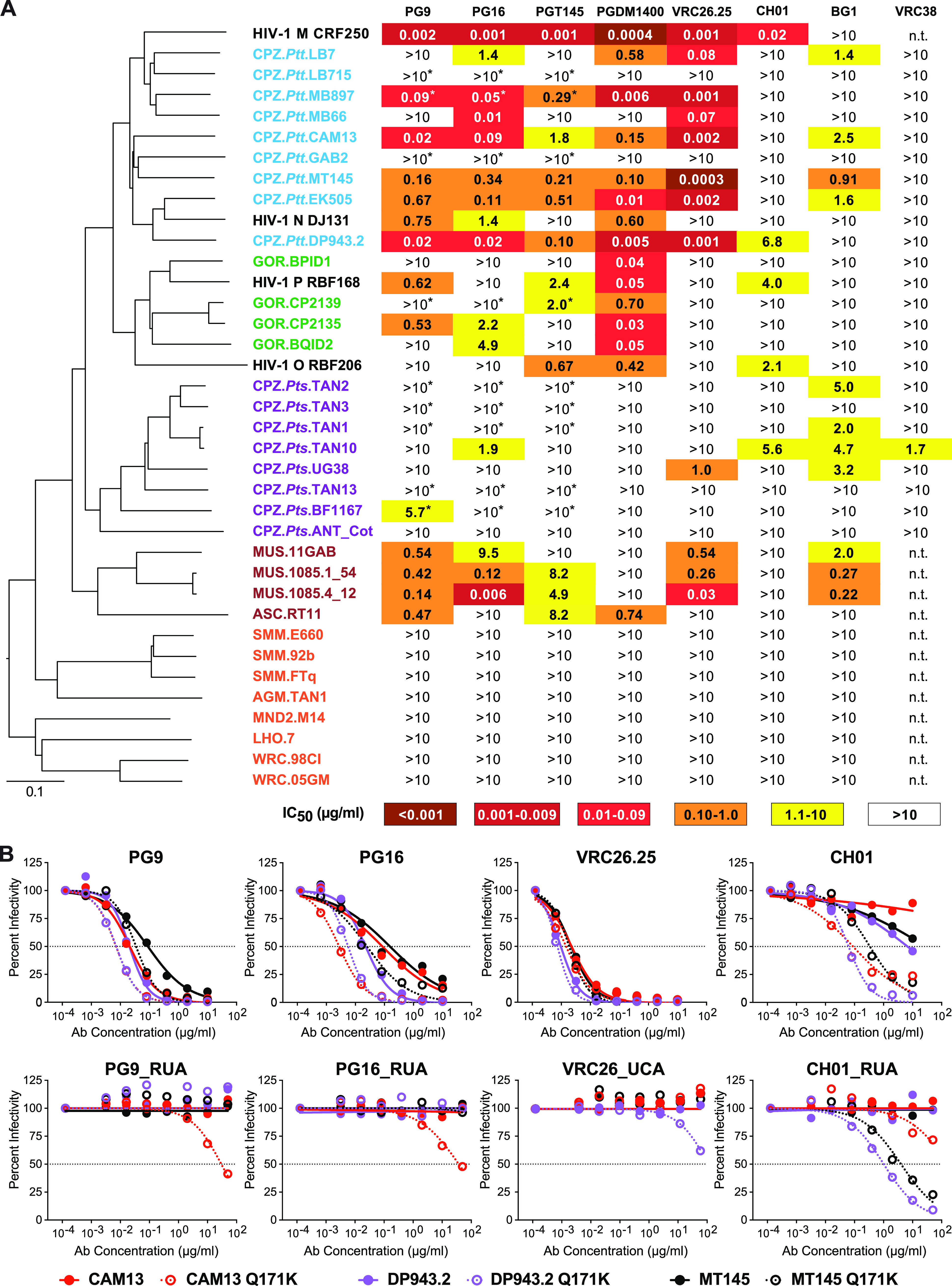
Antigenic conservation of the V2-apex among primate lentiviruses. (A) The ability of human V2-apex bNAbs (top) to neutralize pseudoviruses bearing Envs from diverse SIV strains (left) is shown, with numbers indicating IC_50_ values (μg/mL) as determined in the TZM-bl assay (39). Coloring indicates the relative neutralization potency. Values highlighted by asterisks have previously been reported (19). SIV Envs are color-coded to indicate their species/subspecies origin (central chimpanzees [CPZ.*Ptt*], blue; western gorillas [GOR], green; eastern chimpanzees [CPZ.*Pts*], purple; moustached [MUS] and red-tailed [ASC] monkeys, brown; sooty mangabeys [SMM], African green monkeys [AGM], mandrills [MND2], L’Hoest’s monkeys [LHO], and western red colobus [WRC], red), and their phylogenetic relationships are illustrated by a neighbor joining tree of full-length Env protein sequences to the left (the scale bar indicates 0.1 amino acid substitutions per site). HIV-1 strains CRF250 (group M), DJ131 (group N), RBF168 (group O), and RBF206 (group P) are shown for comparison. The highest antibody concentration used was 10 μg/mL (n.t., not tested). (B) Neutralization curves depicting the sensitivity of pseudoviruses containing wild-type (closed circles) and Q171K mutated (open circles) versions of the SIVcpz Envs CAM13 (red), DP943.2 (blue), and MT145 (black) to mature V2-apex bNAbs PG9, PG16, VRC26.25, and CH01 (top) and their inferred precursors (bottom). Dashed lines indicate 50% reduction in virus infectivity (the antibody concentration is shown on the *x* axis in μg/mL).

SIV Env expression plasmids were cotransfected with an *env*-minus SIVcpz proviral backbone in 293T cells, and the neutralization sensitivity of the resulting pseudoviruses was tested in the TZM-bl assay (39). This analysis identified several SIVcpz and SIVgor Env pseudotypes that were uniquely sensitive to V2-apex bNAbs, with half-maximal inhibitory concentrations (IC_50_) of less than 1 μg/mL (Fig. 1A). The most sensitive Envs (MB897, CAM13, MT145, EK505, DP943), all of which were potently neutralized by PG9, PG16, PGT145, PGDM1400, and VRC26.25, were derived from SIVcpz*Ptt* strains of central chimpanzees (*P. t. troglodytes*), which are the closest relatives of pandemic (group M) HIV-1. Envs from more distantly related gorilla viruses were also sensitive to V2-apex bNAbs, especially to PGDM1400 (IC_50_ <0.7 μg/mL), although they were resistant to VRC26.25, CH01, BG1, and VRC38. Envs from the most distantly related SIVcpz*Pts* strains infecting eastern chimpanzees (P. t. schweinfurthii) were the least sensitive, although four were neutralized by BG1 (IC_50_ <5 μg/mL).

We also tested Envs from primate lentiviruses infecting African monkey species. These analyses showed that some SIVs, such as SIVmus from moustached monkeys and SIVasc from red-tailed monkeys, were sensitive to human V2-apex bNAbs (Fig. 1A). SIVmus Envs were particularly sensitive to PG9, PG16, VRC26.25, and BG1, while the SIVasc Env was potently neutralized by PG9 and PGDM1400 (IC_50_ <1 μg/mL). In contrast, SIVs infecting sooty mangabeys (*Cercocebus atys*), tantalus monkeys (*Chlorocebus tantalus*), mandrills (*Mandrillus sphinx*), l’Hoest’s monkeys (*Allochrocebus lhoesti*), and western red colobus (*Piliocolobus badius*) were resistant to V2-apex bNAbs (Fig. 1A). All of these lacked a potential N-linked glycosylation site (PNGS) at position 156, which is important for most V2 apex bNAbs (36, 40, 41), and contained substitutions in the core V2 epitope consistent with neutralization resistance. Thus, the antigenic conservation of the trimer apex is not limited to the ape precursors of HIV-1 but extends to some SIVs infecting Old World monkeys.

### Germ line-targeting potential of V2-apex bNAb-sensitive SIV Envs.

To examine whether any of the V2-apex bNAb-sensitive SIV Envs had germ line-targeting potential, we tested their neutralization sensitivity to the reverted unmutated ancestors (RUAs) of PG9, PG16, and CH01 (42) and the inferred unmutated common ancestor (UCA) of VRC26 (29). As expected, none of the wild-type SIV Envs were neutralized by the precursor antibodies (Table S1 in the supplemental material). Since a glutamine-to-lysine mutation at position 171 (Q171K) in the MT145 Env had conferred the ability to bind to human V2-apex germ line precursors (20), we tested the effect of this mutation in all other SIV Envs. Moreover, we modified select SIV Envs by introducing additional changes previously reported to improve germ line-targeting of HIV-1 Envs (20, 26), including removing a PNGS at position 130 (N130K), adding a PNGS at position 156 (K156N + F158S), changing a lysine at position 166 to an arginine (K166R), and/or increasing the number of lysine residues in the C strand (Table S1). While most of these mutations increased the neutralization sensitivity of the SIV Envs to the mature bNAbs, this was not true for the corresponding precursors. Of 20 SIVcpz, SIVgor, SIVmus, and SIVasc Env mutants tested, only three were neutralized by the RUAs of PG9, PG16, and/or CH01 at IC_50_ values of <50 μg/mL (Fig. 1B; Table S1). These included the previously reported MT145K Env (20) as well as two new minimally modified Envs, CAM13K and DP943K, from SIVcpz*Ptt* strains originally isolated from naturally infected chimpanzees in Cameroon (43, 44). Both DP943K and MT145K were only neutralized by the CH01_RUA, while CAM13K was neutralized by the RUAs of PG9, PG16, and CH01, although the latter did not reach 50% inhibition (Fig. 1B). Since the CAM13K Env appeared to engage more than one V2-apex bNAb precursor, it was selected for further studies.

10.1128/mbio.03370-22.1 TABLE S1 Neutralization sensitivity of wild-type and modified SIV Envs to V2-apex bNAbs and their inferred precursors. Download Table S1, PDF file, 0.04 MB. Copyright © 2023 Bibollet-Ruche et al. 2023 Bibollet-Ruche et al. https://creativecommons.org/licenses/by/4.0/ This content is distributed under the terms of the Creative Commons Attribution 4.0 International license.

### Immunogenicity of a CAM13K SOSIP trimer in CH01 germ line heavy chain knock-in mice.

To test the germ line-targeting potential of the CAM13K Env, we expressed it as a soluble SOSIP trimer (Fig. S1A) using previously reported stabilization mutations (45 to 48). Biolayer interferometry (BLI) showed that the CAM13K trimer bound conformation-dependent V2-apex, but not the CD4 binding site and interface bNAbs (Fig. S1B). There also was no appreciable binding of nonneutralizing antibodies directed against V2, V3, and CD4-induced epitopes (Fig. S1C). Consistent with the neutralization results (Fig. 1B), the CAM13K SOSIP trimer bound the RUAs of PG9, PG16, and CH01 (Fig. S1B and C). Finally, negative stain electron microscopy (NSEM) showed a well-ordered trimer (Fig. S1D), suggesting a native-like Env configuration.

10.1128/mbio.03370-22.2 FIG S1 Generation and characterization of a CAM13K SOSIP trimer. Download FIG S1, PDF file, 0.6 MB. Copyright © 2023 Bibollet-Ruche et al. 2023 Bibollet-Ruche et al. https://creativecommons.org/licenses/by/4.0/ This content is distributed under the terms of the Creative Commons Attribution 4.0 International license.

**FIG 2:**
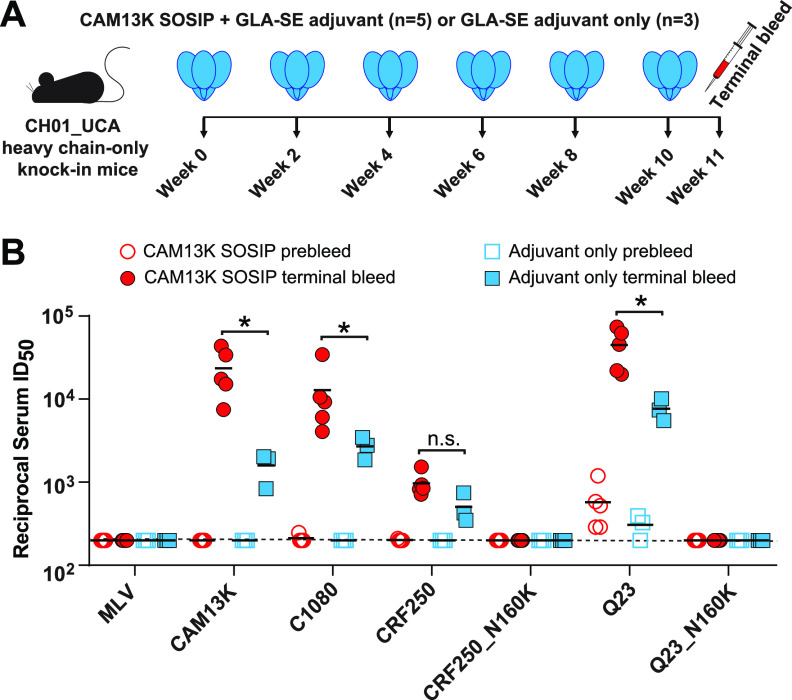
CAM13K SOSIP immunization elicits neutralizing antibody responses in V2-apex bNAb precursor expressing knock-in mice. (A) CH01_RUA heavy chain (HC)-only knock-in mice (20) were immunized either with 20 μg CAM13K SOSIP trimer in 5 μg GLA-SE adjuvant (*n* = 5) or with 5 μg GLA-SE adjuvant alone (*n* = 3). Time points of immunization and bleeds are indicated. (B) Comparison of serum neutralization titers (reciprocal 50% inhibitory dilutions, ID_50_) from trimer-immunized (red circles) and adjuvant-only (blue squares) knock-in mice (preimmunization and terminal bleeds are shown as open and filled symbols, respectively). Serum neutralizing activity was tested in the TZM-bl assay (39) against pseudoviruses carrying the immunogen-matched autologous CAM13K Env, three CH01_RUA sensitive HIV-1 Envs (C1080, CRF250, Q23), as well as N160 glycan knockout variants of CRF250 and Q23. MLV Env containing pseudovirus was used for control. Differences between immunized and adjuvant-only groups were assessed using a nonparametric Mann-Whitney test (Prism 9.4.0 GraphPad Software), with asterisks indicating *P* < 0.05.

To determine whether the CAM13K trimer could activate human V2-apex bNAb precursor-expressing B cells *in vivo*, we tested it in a previously described knock-in mouse (20). In this model, animals are homozygous for the prearranged heavy chain (V_H_DJ_H_^+/+^) of the inferred precursor of the V2-apex bNAb CH01, which then pairs with wild-type mouse light chains (20). Five mice were immunized with the CAM13K SOSIP trimer in glucopyranosyl lipid adjuvant-stable emulsion (GLA-SE), while three mice received only GLA-SE. After six immunizations, mice were necropsied and terminal bleeds tested for neutralizing activity (Fig. 2). Although adjuvant administration alone elicited some serum neutralization (reflecting the high precursor frequency), SOSIP immunization induced significantly higher titers to both autologous (CAM13K) and heterologous (C1080, CRF250, Q23) pseudoviruses, which were neutralized in an N160 glycan-dependent manner (Fig. 2). Since the three HIV-1 Envs are sensitive to neutralization by the CH01_RUA (Fig. S2), these findings do not indicate the development of heterologous breadth. However, the data show that a soluble CAM13K Env trimer was able to expand human V2-apex bNAb precursor-encoding mouse B cells and induce a V2-apex directed neutralization response.

10.1128/mbio.03370-22.3 FIG S2 Sensitivity of HIV-1 Env pseudoviruses to neutralization by CH01_RUA. Download FIG S2, PDF file, 0.1 MB. Copyright © 2023 Bibollet-Ruche et al. 2023 Bibollet-Ruche et al. https://creativecommons.org/licenses/by/4.0/ This content is distributed under the terms of the Creative Commons Attribution 4.0 International license.

### SCIV induced autologous and heterologous NAb responses in RMs.

**FIG 3:**
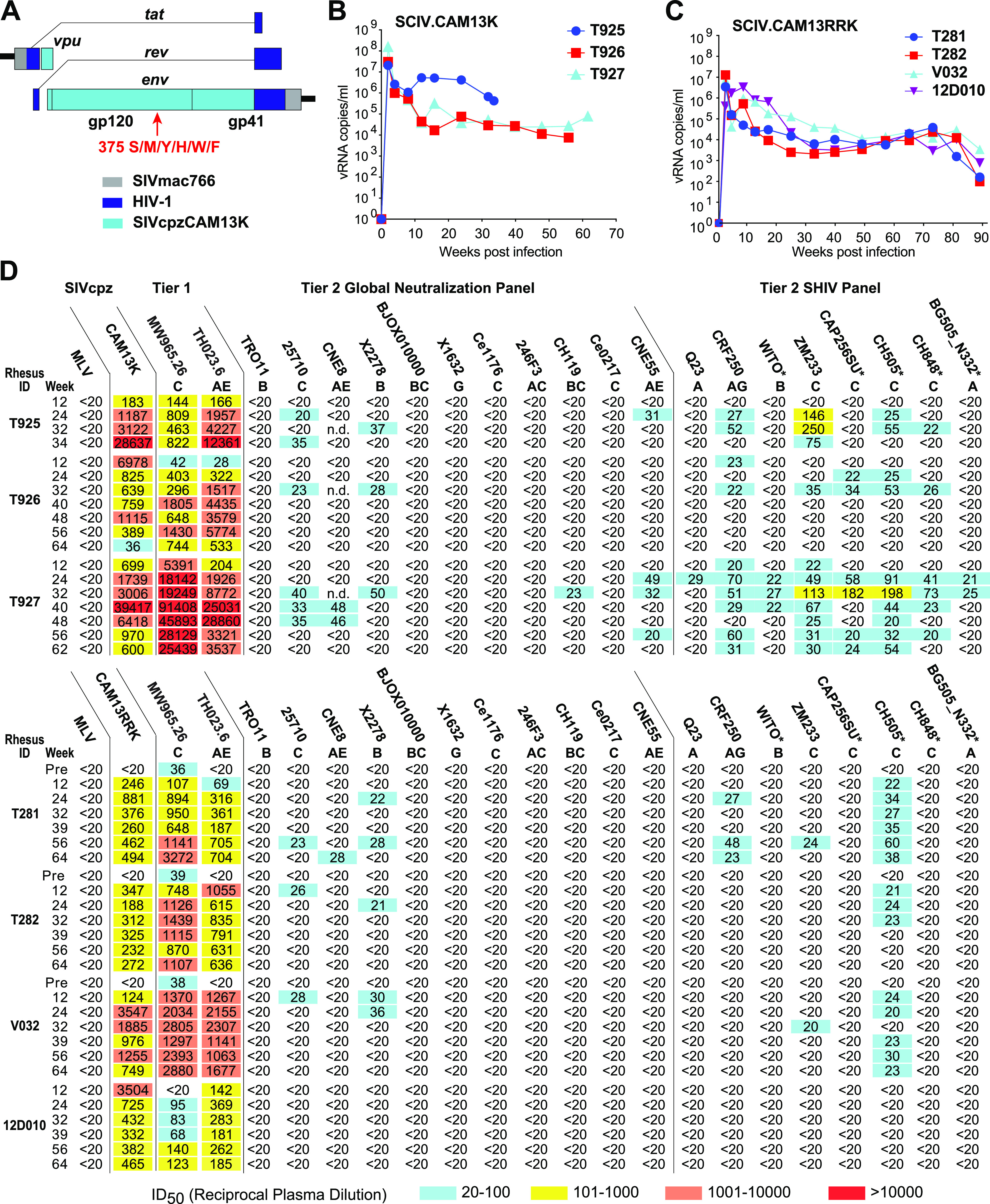
SCIV-infected RMs develop low-titer heterologous neutralization breadth. (A) Design scheme of SCIV vectors expressing SIVcpz Env ectodomains. The SIVcpz CAM13K *vpu-env* region (teal) was cloned into an optimized SHIV vector (21) consisting of a SIVmac766 proviral backbone (gray) and HIV-1-derived *tat* and *rev* genes (dark blue). Six isogenic SCIV mutants were generated with serine (S), methionine (M), tyrosine (Y), histidine (H), tryptophan (W) or phenylalanine (F) at position 375 of the CAM13K Env. (B) Plasma vRNA kinetics in three RMs infected with SCIV.CAM13K. Animals were inoculated with equal mixtures of SCIV.CAM13K variants bearing all six Env375 mutants. SCIV.CAM13K.M375W emerged as the predominant strain in all animals. (C) Plasma vRNA kinetics in four RMs infected SCIV.CAM13RRK (generated from SCIV.CAM13K.375W by introducing K169R and K170R mutations). (D) Autologous and heterologous neutralization of longitudinal plasma samples from RMs infected with SCIV.CAM13K (top) or SCIV.CAM13RRK (bottom). Reciprocal 50% inhibitory dilutions (ID_50_) are shown for autologous (wild-type CAM13K and CAM13RRK encoding 375M) and heterologous (tier 1 and tier 2) viruses representing different HIV-1 subtypes (A, AG, AE, AC, B, C, BC, G; indicated below virus name), with no reactivity observed against a murine leukemia virus (MLV) Env containing control (all ID_50_ <1:20). Coloring indicates relative neutralization potency. Both Env pseudotypes (CAM13K, CAM13RRK, tier 1 and tier 2 global panel) and replication-competent SHIV strains were tested, all of which encoded the wild-type amino acid at position 375, except for SHIV.BG505_N332, which encoded a tyrosine instead of a serine. SHIVs expressing transmitted founder HIV-1 Envs are denoted by asterisks. For three SCIV.CAM13RRK-infected animals, which were repurposed from prior HIV-1 immunization studies (see methods), preinfection (pre) plasma neutralization titers are also shown.

**FIG 4:**
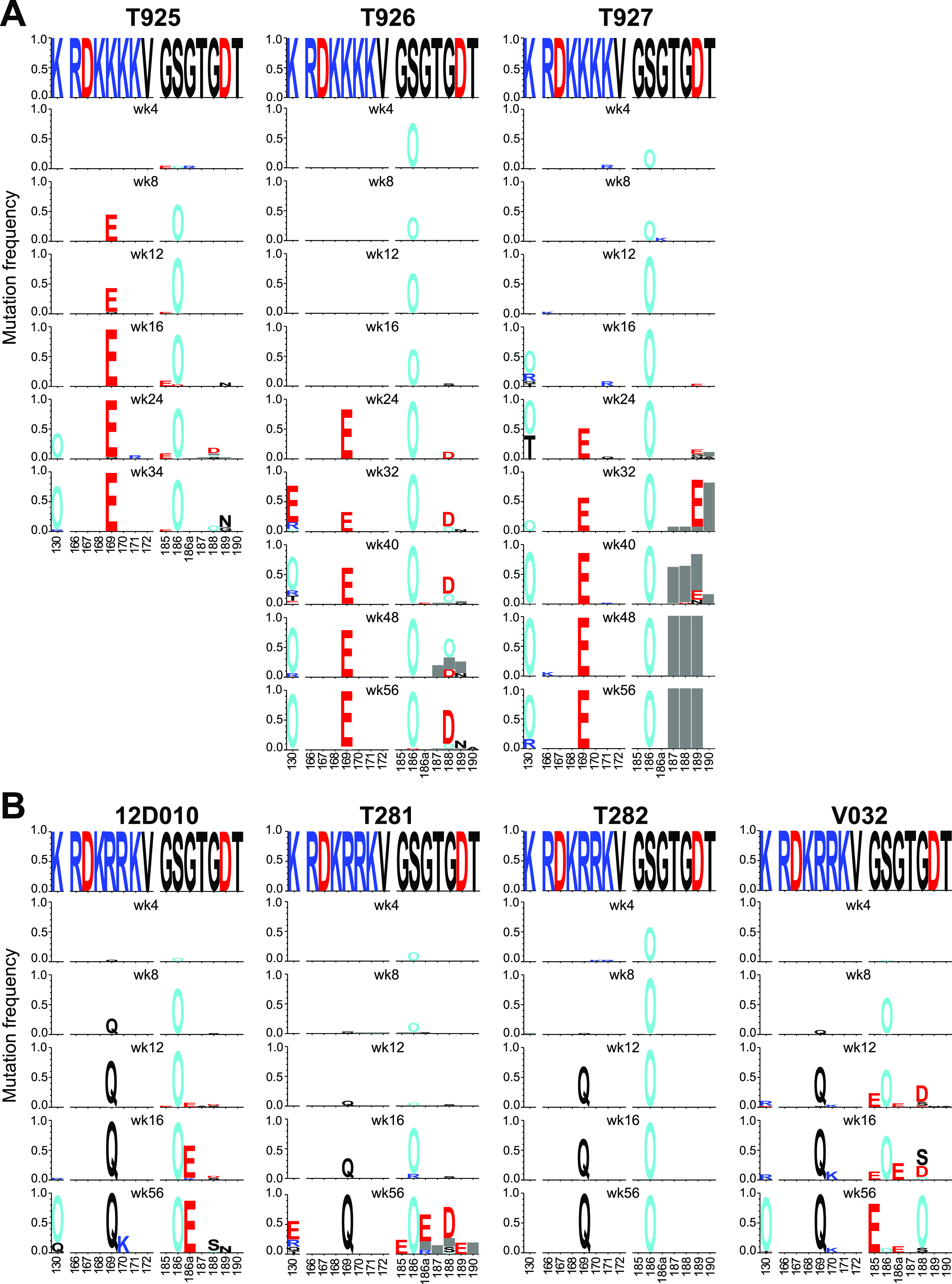
SCIV infection elicits strong V2-apex directed immune selection. (A, B) Longitudinal Env evolution at the V2-apex is shown over time for the N-linked glycosylation site at position 130, strand C (amino acids 166 to 172), and the hypervariable part of the V2 loop (amino acids 185 to 190) for all SCIV.CAM13K (A) and SCIV.CAM13RRK (B) infected RMs (see Fig. S3 for a similar analysis of the entire Env). Sequences are shown as logo plots, where the height of each amino acid is proportional to its frequency at the respective time point. For each RM, the top logo depicts the infecting SCIV, with subsequent logos representing longitudinal time points denoted in weeks (wk). To highlight mutations, the infecting virus sequence is blanked out for all longitudinal time points. Positively charged amino acids (R, H, K) are colored blue, negatively charged amino acids (D, E) are colored red, potential N-linked glycosylation sites are denoted as “O” and colored cyan, and gray bar indicate deletions. The webtool AnalyzeAlign from the Los Alamos HIV Databases was used for to generate the logos.

SHIV infections provide insight into the immunogenicity of HIV-1 Env glycoproteins since these viruses express native trimers that coevolve with germ line and intermediate B cell receptors, thereby driving somatic hypermutation and antibody maturation (12). To test the bNAb induction potential of the CAM13K Env in the context of a productive viral infection, we cloned its ectodomain (Fig. 3A) into an SIVmac766 vector previously optimized for SHIV construction (21). Since the amino acid residue at position 375 of the HIV-1 Env determines how efficiently the corresponding SHIV replicates in rhesus CD4^+^ T cells (21, 49), we created isogenic mutants of SCIV.CAM13K by changing the wild-type methionine (375M) to serine (375S), tyrosine (375Y), histidine (375H), tryptophan (375W), or phenylalanine (375F). These constructs were used as equal mixtures (based on p27 content) to infect three naive RMs by intravenous inoculation (Fig. 3B). Single genome sequencing of plasma viral RNA 4 weeks postinfection identified the 375W variant as the predominant strain in all three animals (Fig. S3). Based on previous signature analyses (50), we also replaced two lysine residues at positions 169 and 170 in the C strand of the CAM13K Env with arginine residues, which markedly enhanced neutralization by the reverted unmutated ancestors of PG9, PG16, and CH01 (Fig. S4). By introducing these same mutations into the 375W variant of SCIV.CAM13K, we generated a second germ line-targeting SCIV.CAM13RRK strain and infected four additional (repurposed) RMs (see Materials and Methods for details). All seven animals were treated with anti-CD8 monoclonal antibodies to transiently deplete CD8^+^ T cells at the time of inoculation and then followed longitudinally for up to 88 weeks to assess viral replication, neutralizing responses, and Env sequence evolution (Fig. 3 and 4).

10.1128/mbio.03370-22.4 FIG S3 Env sequence evolution in SCIV-infected rhesus macaques. Download FIG S3, PDF file, 0.6 MB. Copyright © 2023 Bibollet-Ruche et al. 2023 Bibollet-Ruche et al. https://creativecommons.org/licenses/by/4.0/ This content is distributed under the terms of the Creative Commons Attribution 4.0 International license.

10.1128/mbio.03370-22.5 FIG S4 Sensitivity of CAM13RRK to neutralization by V2-apex bNAb precursors. Download FIG S4, PDF file, 0.2 MB. Copyright © 2023 Bibollet-Ruche et al. 2023 Bibollet-Ruche et al. https://creativecommons.org/licenses/by/4.0/ This content is distributed under the terms of the Creative Commons Attribution 4.0 International license.

Both SCIV.CAM13K and SCIV.CAM13RRK established productive and persistent infections in all animals (Fig. 3B and C). Although there was a trend for higher peak (geometric mean [GM] 4.6 × 10^7^ versus 2.7 × 10^6^ vRNA/mL at week 2) and setpoint (GM 2.3 × 10^5^ versus 1.4 × 10^4^ vRNA/mL at week 24) viral loads in SCIV.CAM13K compared to SCIV.CAM13RRK-infected animals, the group sizes were too small to reliably estimate differences. One SCIV.CAM13K-infected RM (T925) with a particularly high set point viral load developed an AIDS-like illness and was euthanized at week 32. All SCIV-infected animals developed potent autologous neutralizing antibodies against wild-type (375M) CAM13K and CAM13RRK pseudoviruses (Fig. 3D).

We also assessed heterologous plasma neutralization using tier 1 (*n* = 2) and tier 2 (*n* = 19) HIV-1 strains, which included global panel pseudoviruses (51) as well as replication-competent SHIV strains (12). Plasma samples from all animals potently neutralized the two tier 1 strains, which are known to spontaneously adopt an open Env conformation (Fig. 3D). Plasma samples from six animals also neutralized up to 11 (of 19) heterologous tier 2 strains, including several transmitted founder Env-expressing SHIVs, which are usually neutralization resistant (52). These heterologous plasma responses were observed very early in infection and mapped to the V2-apex epitope, since mutations at positions 166, 169, or 171 in susceptible Envs reduced their neutralization sensitivity (Fig. S5). Surprisingly, however, all heterologous plasma responses were only weakly cross-neutralizing, with most reciprocal 50% inhibitory dilutions (ID_50_) just above the 1:20 cutoff. Moreover, there was no appreciable increase in breadth and potency over time (Fig. 3D), as is typically seen in SHIV-infected RMs or HIV-1-infected humans who develop canonical bNAbs. Thus, SCIV infection elicited atypical V2-directed neutralizing antibodies that did not represent V2 bNAbs.

10.1128/mbio.03370-22.6 FIG S5 Epitope mapping of plasma-neutralizing antibodies in SCIV-infected RMs. Download FIG S5, PDF file, 0.3 MB. Copyright © 2023 Bibollet-Ruche et al. 2023 Bibollet-Ruche et al. https://creativecommons.org/licenses/by/4.0/ This content is distributed under the terms of the Creative Commons Attribution 4.0 International license.

### Env sequence evolution in SCIV-infected rhesus macaques.

To look for evidence of immune selection, we characterized the evolving Env quasispecies in all SCIV-infected RMs. This was done by limiting dilution PCR of plasma viral RNA, in which sequencing of single RNA templates allowed the tracking of sequence changes from the transmitted virus over time (Fig. S3). Longitudinal sequences were analyzed using computational tools designed to investigate different aspects of within-host Env evolution. One of these, termed LASSIE (53), identified Env residues where mutations altered the encoded amino acid in at least 80% of sequences at one or more time points. This approach identified several residues at the V2-apex that exhibited signs of immune selection (Fig. 4).

The first mutation observed in all animals by week 4 involved the addition of a PNGS at position 186 (S186N) in the V2 loop (Fig. 4). This was followed as early as week 8 by changes at position 169 in the C strand, which is a known contact residue of V2-apex bNAbs (30, 32, 40, 54, 55). In all SCIV.CAM13K-infected RMs, a lysine at this position was changed to a glutamic acid (K169E) (Fig. 4A), while in all SCIV.CAM13RRK-infected RMs an arginine was changed to a glutamine (R169Q), in each case requiring a single nucleotide mutation (Fig. 4B). A third mutation that emerged as early as week 16 was a K130N substitution, which resulted in the addition of an N-linked glycosylation site at position 130. This change was observed in 5 of the 7 SCIV-infected animals, and like the changes at position 169, confers resistance to most V2-apex bNAbs (30, 32, 40, 50, 54, 55). Although these sites were not the only ones displaying signs of selection, they were among the first to change in all, or nearly all, SCIV-infected animals, indicating escape from strong V2-directed antibody pressures.

Since glycan holes can detract from bNAb development (56), we investigated the evolution of the glycan shield in the SCIV-infected RMs. We found that CAM13K and CAM13RRK Envs have five strain-specific glycan holes, three of which were filled during the infection course (Fig. S6A). We also examined Env hypervariable loops, which evolve primarily by insertions and deletions and are thus difficult to align. Using alignment-free characteristics such as length, number of glycans, and net charge, we found very similar patterns of variable loop evolution across all RMs (Fig. S6B). The most notable changes were deletions in the hypervariable part of V1 (HXB2 positions 132 to 152), which is exceptionally long in CAM13 (31 AA) and decreased by as many as 14 amino acids. This reduction in V1 loop size was accompanied by a loss of one or two glycosylation sites as well as a decrease in net charge (Fig. S6B). There also was a trend of a net charge reduction in the V2 hypervariable loop, but this was not observed in all animals. In contrast, no or only minor changes in length, glycan number, and net charge were observed for variable loops 4 and 5. Since long, negatively charged V1 and V2 loops are associated with resistance to V2-apex bNAbs (50), these findings provided further evidence for a strong V2-directed antibody response.

10.1128/mbio.03370-22.7 FIG S6 Glycan shield and hypervariable loop evolution in SCIV-infected RMs. Download FIG S6, PDF file, 1.8 MB. Copyright © 2023 Bibollet-Ruche et al. 2023 Bibollet-Ruche et al. https://creativecommons.org/licenses/by/4.0/ This content is distributed under the terms of the Creative Commons Attribution 4.0 International license.

### Cloning of antigen-specific B cells from SCIV-infected rhesus macaques.

To characterize further the SCIV-induced plasma breadth, we selected RMs T925 and T927 for single B cell sorting. Both animals were SCIV.CAM13K-infected, not previously immunized, and exhibited the highest plasma neutralization titers (Fig. 3D). Matching the probes to the plasma neutralizing activity, we used ZM197-ZM233 (20) and CAP256 (55) SOSIP hooks to isolate antigen-specific B cells. For RM T927, we used both probes to sort peripheral blood mononuclear cells (PBMCs) collected 24 and 32 weeks postinfection but only the ZM197-ZM233 SOSIP to sort lymph node cells obtained at necropsy 62 weeks postinfection. For RM T925, we used the ZM197-ZM233 SOSIP to sort B cells from PBMCs obtained 24 weeks postinfection. We identified multiple expanded lineages in both animals and selected seven for paired heavy and light chain immunoglobulin gene amplifications based on V2-apex bNAb-like HCDR3 lengths and D gene usage (Table 1). Select lineage members were then tested for neutralization against the same panel of tier 1 and tier 2 HIV-1 and SHIV strains used to characterize the plasma samples.

**TABLE 1:** Immunogenetics of Env-specific monoclonal antibodies from SCIV-infected rhesus macaques

RM	Expanded lineage	No of Members	Wks post infection	HDCR3 length (AA)	IGHV gene^*a*^	% HV somatic hypermutation	IGHD gene^*a*^	IGHJ gene^*a*^	IGLV gene^*b*^	IGLJ gene^*b*^	Autologous neutralization	Heterologous neutralization	
												HIV-1	SIVcpz
T927	L1	3	32	24	IGHV4-79*02_S9501	5.1	IGHD3-9*01	IGHJ6-6*01	IGLV3-40*01	IGLJ2A*01	Yes	0/19	0/1
	L2	19	24	15	IGHV5-15*01_S2502	4.2 to 5.8	IGHD6-13*01	IGHJ1*02	IGLV2S9*01	IGLJ1*01	Yes	1/19	0/1
	L3	3	62	27	IGHV4-149*01_S1992	5.1 to 6.5	IGHD3-9*01	IGHJ5-5*01	IGLV1-85*01	IGLJ2A*01	No	2/19	0/1
	L4	9	62	27	IGHV4-149*01_S1992	6.9 to 9.0	IGHD3-9*01	IGHJ4-3*01	IGLV1-67*02	IGLJ3*01	Yes	5/19	1/1
	L5	7	24, 62	22	IGHV3-50*01	4.4 to 14.2	IGHD3-9*01	IGHJ5-4*03	IGKV2-104*02	IGKJ1*01	Yes	6/19	1/1
T925	L1	5	24	18	IGHV4-117*01_S5847	1.4 to 4.4	IGHD3-9*01	IGHJ4-3*01	IGKV1-94*01	IGKJ4*01	Yes	7/19	1/1
	L2	27	24	18	IGHV4-NL_33*01_S4467	4.0 to 7.7	IGHD3-18*01	IGHJ6-6*01	IGLV2-23*02	IGLJ3*01	No	0/2	n.d.^*c*^

a Rhesus macaque heavy (H) chain variable (V), diversity (D), and joining (J) gene names are from Bernat et al., 2021 (57), except for the D genes of T927 L1-5 and T925 L1, and the J gene of T927 L2, which are from the International ImMunoGeneTics Information System (IMGT) (see Table S2E at https://doi.org/10.6084/m9.figshare.21644675 for the GenBank accession numbers of the corresponding nucleotide sequences).

b Rhesus macaque light (L) chain variable (V) and joining (J) gene names are from IMGT.

c n.d., not done.

**FIG 5:**
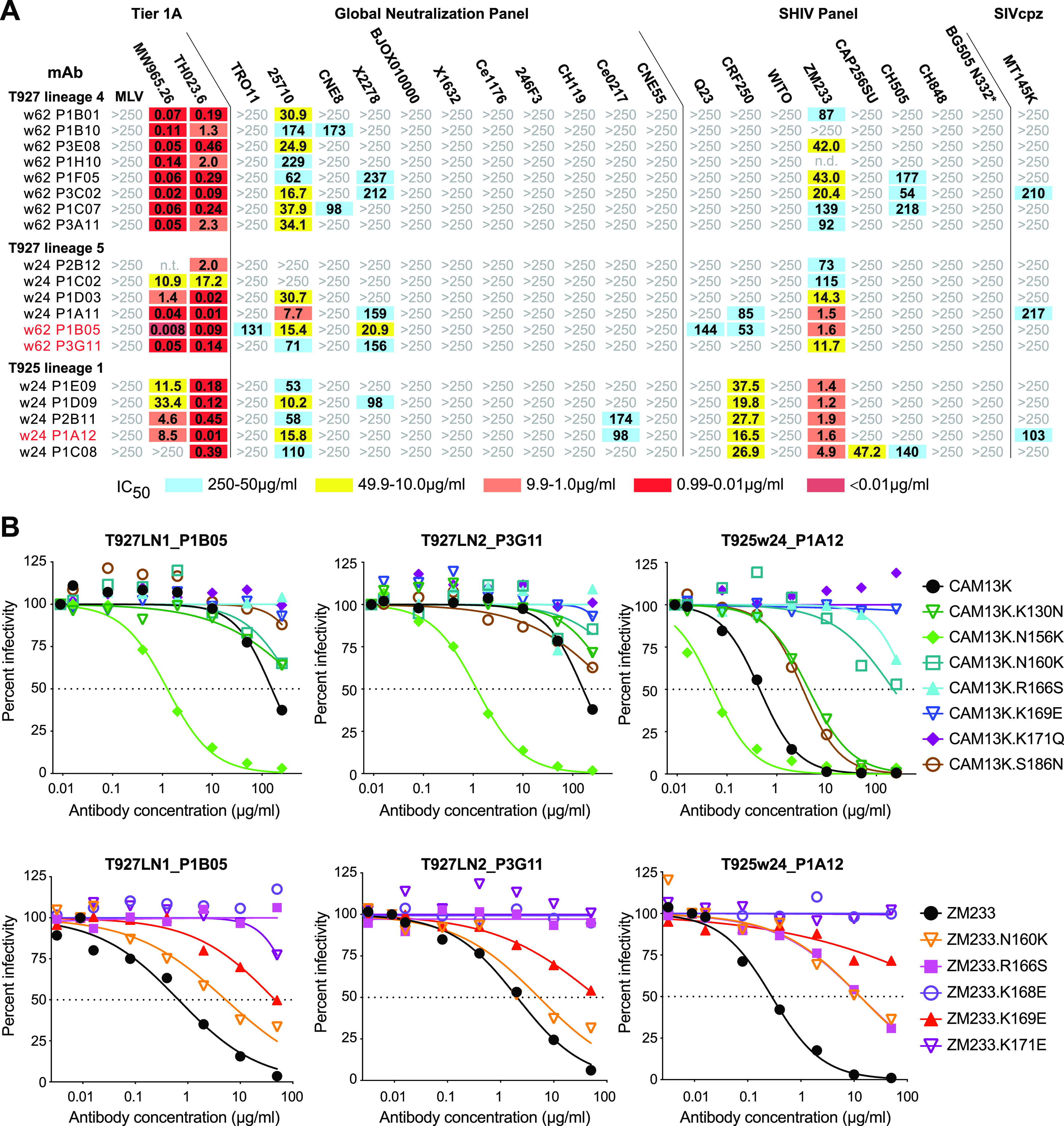
SCIV infections elicit low-potency V2-directed antibodies that neutralize HIV-1. (A) Neutralization breadth and potency of SCIV-induced antibodies cloned from RMs T927 and T925 (indicated on the left). Fifty percent inhibitory concentrations (IC_50_) are shown for representative lineage members (μg/mL) against pseudoviruses and SHIV strains (indicated on top). Only the most potent and cross-reactive lineages are shown (see Fig. S7A for similar results for the remaining lineages). The highest antibody concentration used was 250 μg/mL (coloring indicates relative neutralization potency; n.t., not tested). MAbs analyzed by negative stain electron microscopy are highlighted in red (heavy and light chain variable region sequences are shown in Fig. S8). (B) Epitope mapping of select cross-neutralizing MAbs. Neutralization curves are shown for three monoclonal antibodies (indicated on top and highlighted in red in panel A) against a panel of CAM13K and ZM233 mutant pseudoviruses (indicated on the right). Dashed lines indicate 50% reduction in virus infectivity (corresponding IC_50_ values are shown in Fig. S7B).

Of the five expanded lineages identified in RM T927, three exhibited no or very limited heterologous activity, with lineage 1 neutralizing no tier 2 HIV-1, lineage 2 neutralizing only ZM233, and lineage 3 neutralizing ZM233 and 25710, but not the autologous CAM13K strain (Fig. S7A). However, the remaining two lineages exhibited greater heterologous breadth, with lineage 4 neutralizing as many as 5 of 19 (26%) and lineage 5 as many as 6 of 19 (32%) tier 2 HIV-1 strains (Table 1; Fig. 5A). The viruses neutralized by both lineages were 25710, X2278, and SHIV.ZM233, although very high antibody concentrations were required to achieve 50% inhibition. One member of each lineage also weakly neutralized the SIVcpz strain MT145K (IC_50_ >200 μg/mL). Antibody isolation from the second animal T925 yielded very similar results. Of the two expanded lineages, only lineage 1 exhibited heterologous breadth, with all members neutralizing 25710, SHIV.CRF250, and SHIV.ZM233, and a subset also neutralizing X2278, CE0217, SHIV.CAP256SU, SHIV.CH505, and SIVcpz.MT145K strains, again with low potency (Fig. 5A). Surprisingly, almost all lineage members neutralized one or both tier 1 viruses with high potency, suggesting that they target conserved epitopes in incompletely closed HIV-1 Env trimers (Fig. 5A). Overall, the isolated antibodies accounted for most of the heterologous plasma activity, although their potency was universally low. This was the case despite several features characteristic for V2-apex bNAbs, such as long HCDR3s (18 to 27 AA), up to 14% heavy chain variable (V) region somatic hypermutation, and the usage of the heavy chain diversity (D) gene HD3-9*01 (also known as HD3-15*01) (57, 58), which encodes an EDDY motif predicted to be tyrosine sulfated (59) (Fig. S8).

10.1128/mbio.03370-22.8 FIG S7 Neutralization properties of antibody lineages isolated from SCIV-infected RMs. Download FIG S7, PDF file, 0.5 MB. Copyright © 2023 Bibollet-Ruche et al. 2023 Bibollet-Ruche et al. https://creativecommons.org/licenses/by/4.0/ This content is distributed under the terms of the Creative Commons Attribution 4.0 International license.

10.1128/mbio.03370-22.9 FIG S8 Heavy and light chain variable region sequences of antibody lineages isolated from SCIV-infected RMs. Download FIG S8, PDF file, 0.7 MB. Copyright © 2023 Bibollet-Ruche et al. 2023 Bibollet-Ruche et al. https://creativecommons.org/licenses/by/4.0/ This content is distributed under the terms of the Creative Commons Attribution 4.0 International license.

Epitope mapping was conducted using autologous (CAM13K) and heterologous (ZM233 and MW965.26) Env mutants, focusing on amino acid substitutions in the central V2 loop. Testing only members of the most potent lineage in each animal, we found that substitutions at positions 166 (R166S), 168 (K168E), 169 (K169E), and/or 171 (K171E, K171Q) either reduced or abrogated neutralization of autologous and heterologous viruses (Fig. 5B; Fig. S7B). Similarly, removal of the PNGS at position 160 (N160K) increased resistance of most lineage members, although this mutation did not completely abrogate neutralization. Addition of glycosylation sites at positions 130 (K130N) and 186 (S186N), which represented early escape mutations in SCIV-infected RMs (Fig. 4), also reduced neutralization, but had an overall only modest effect (Fig. S7B). Surprisingly, removal of the PNGS at position 156 (N156K), which is required by most V2-apex bNAbs (36, 40, 41), rendered CAM13K up to 2 orders of magnitude more sensitive to neutralization (both ZM233 and MW965.26 lack the N156 glycan). This was observed for all lineages, even the much less potent and cross-reactive ones that failed to neutralize the autologous CAM13K strain (Fig. S7B). Thus, the SCIV-induced cross-neutralizing antibodies targeted the V2 loop, but in a manner distinct from prototypic V2-apex bNAbs.

### Epitope mapping by negative stain electron microscopy.

**FIG 6:**
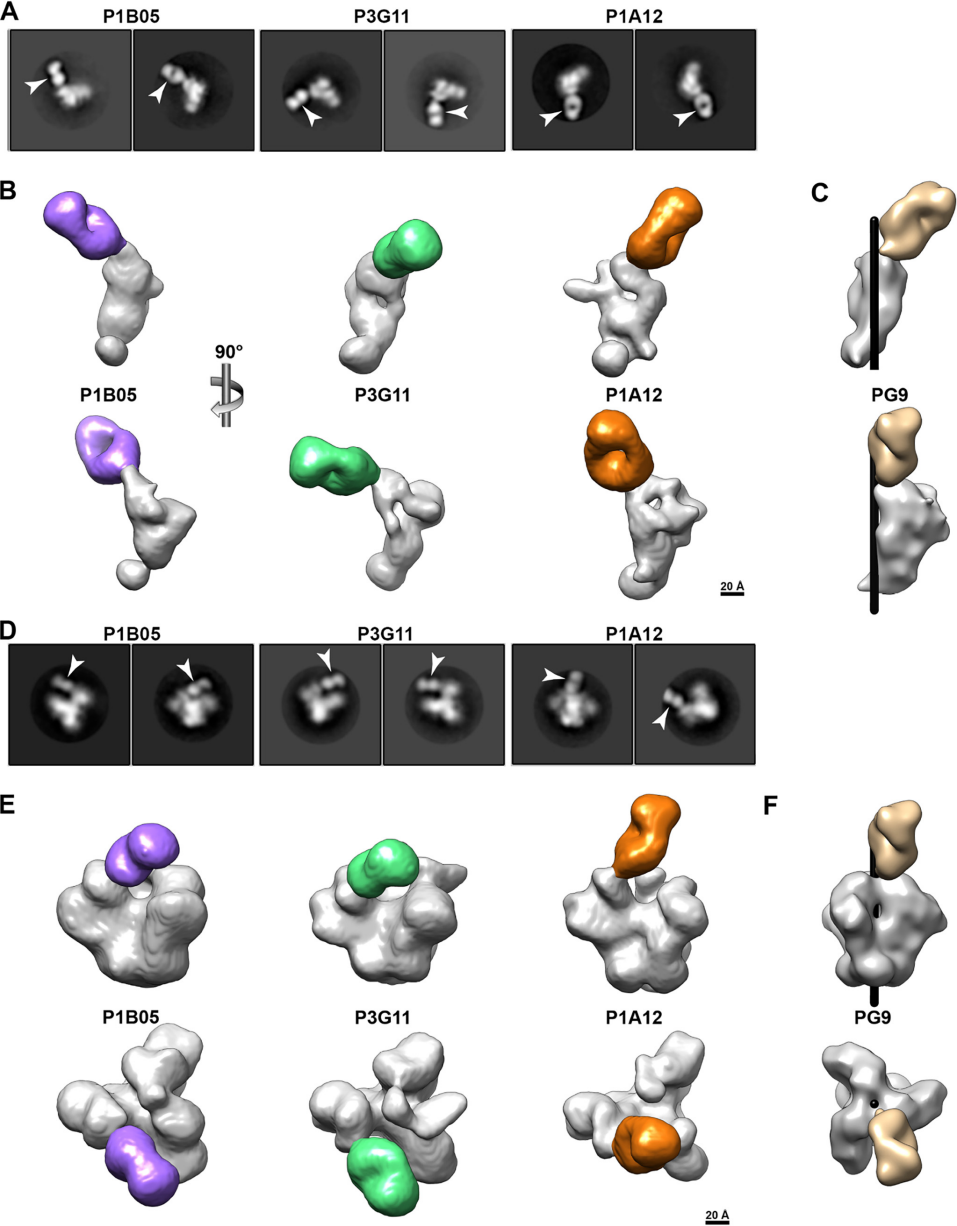
SCIV-induced V2-directed cross-neutralizing antibodies bind occluded-open but not closed trimers. (A) 2D class averages of NSEM images showing Fabs (arrows) bound to single Env protomers. Fabs (denoted on top) were generated for members of the most cross-reactive antibody lineage from RM T927 (P3G11 and P1B05) and RM T925 (P1A12), respectively (also see Fig. 5). Although both ZM197-ZM233 and CAM13K SOSIP preparations were used, only Fab-monomer complexes were observed. (B) 3D reconstructions of Fab-monomer complexes, shown in two orthogonal views, with the Env domain in gray and the Fab in color (P1B05, purple; P3G11, green; P1A12, gold). The scale bar indicates 20 Å. (C) Volume rendering of the PG9 Fab (tan, PDB 3U2S) in complex with a prefusion BG505 trimer (PDB 5FYL), of which only a single protomer (gray) is shown. The Env densities in panel B were aligned with the Env density in panel C and are shown in the same orientation. The black cylinder denotes the position of the 3-fold trimer axis. (D) 2D class averages showing Fabs (arrows) bound to SOSIP trimers lacking the N156 glycan (CH505_N156Q SOSIP). (E) 3D reconstructions of Fabs bound to CH505_N156Q SOSIP trimers. Both side (upper) and top (lower) views are shown, with the Fabs highlighted in color as in panel B. The densities of the CH505_N156Q SOSIP are consistent with an occluded-open trimer. The scale bar indicates 20 Å. (F) Volume rendering of PG9 (tan, PDB 3U2S) in complex with a closed, prefusion BG505 trimer (PDB 5FYL), shown in the same orientation as in panel E. The black cylinder indicates the central 3-fold axis of the Env trimer.

To characterize their modes of interaction, we generated Fab fragments for members of the two most cross-reactive lineages (P1B05 and P3G11 for T927_L5, and P1A12 for T925_L1) and analyzed their binding to both CAM13K and ZM197-ZM233 SOSIP trimers using NSEM. In all attempts to visualize Fab-Env complexes, we failed to observe SOSIP binding of any of the three Fabs, with most two-dimensional (2D) class averages showing trimers with no Fabs bound (Fig. S9, panels A, G, and M). However, a smaller fraction of particles appeared to consist of single Fabs bound to single gp120/gp41 protomers (Fig. 6A), which allowed us to generate three-dimensional (3D) reconstructions (Fig. 6B). For each Fab, we were thus able to infer the binding site, which identified a region in the V2 loop near glycans N156 and N160 (Env positions 158 to 172) as the likely epitope (Fig. S9, panels B, H, and N). However, unlike prototypical V2-apex bNAbs, all three Fabs used an angle of approach that when modeled onto a closed, prefusion trimer resulted in clashes with adjacent protomers. P1B05 and P3G11 used a more horizontal angle of approach compared to canonical bNAbs (Fig. S9, panels C and I), while P1A12 bound close to the trimer axis similar to PG9 (Fig. 6C) but appeared to be rotated, thereby resulting in clashes with neighboring protomers (Fig. S9, panel O). Importantly, such clashes were not observed when Fab binding was modeled onto an occluded-open trimer (Fig. S9, panels D, J, and P), in which the protomers are rotated away from the central axis, but the V1-V3 loops are still in their closed position, shielding the coreceptor binding site (27, 28). Thus, the SCIV-induced cross-neutralizing antibodies either required or induced an occluded-open Env trimer.

10.1128/mbio.03370-22.10 FIG S9 Negative stain EM analysis of SCIV-induced cross-neutralizing antibodies. Download FIG S9, PDF file, 0.5 MB. Copyright © 2023 Bibollet-Ruche et al. 2023 Bibollet-Ruche et al. https://creativecommons.org/licenses/by/4.0/ This content is distributed under the terms of the Creative Commons Attribution 4.0 International license.

The NSEM data suggested that the SCIV-induced antibodies bound Env trimers that were neither completely open (60) nor completely closed (61). To demonstrate this directly, we performed NSEM using an N156 glycan-deficient SOSIP trimer. We reasoned that such a trimer was more likely to be partially open since the absence of the N156 glycan is known to reduce inter-V2-strand interactions and apex stability (41). Moreover, all SCIV-induced cross-reactive NAbs neutralized N156-deficient CAM13K with markedly increased potency (Fig. S7B). We thus examined the binding of the three Fabs to a CH505.N156Q SOSIP trimer. In contrast to all previous attempts, this SOSIP yielded Fab-trimer complexes that were readily visualized in 2D class averages (Fig. 6D). Corresponding 3D reconstructions revealed Fab binding to the V2 region, with the overall trimer density consistent with an occluded-open (Fig. 6E) but not a closed (Fig. 6F) conformation. Importantly, the Fab-monomer structures fit well into the corresponding Fab-trimer structures (Fig. S9, panels E, K, and Q), thus validating their utility to infer putative epitopes and approximate angles of approach. Indeed, the Fab-trimer structures suggested the same epitopes as identified by the Fab-monomers (Fig. S9, panels F, L, and R). Thus, weakly cross-neutralizing antibodies from two different SCIV-infected rhesus macaques bound to a cryptic V2 epitope that appeared to only be accessible in an occluded-open trimer conformation.

### Probing SCIV-expressed and wild-type Envs with conformation-sensitive antibodies.

**FIG 7:**
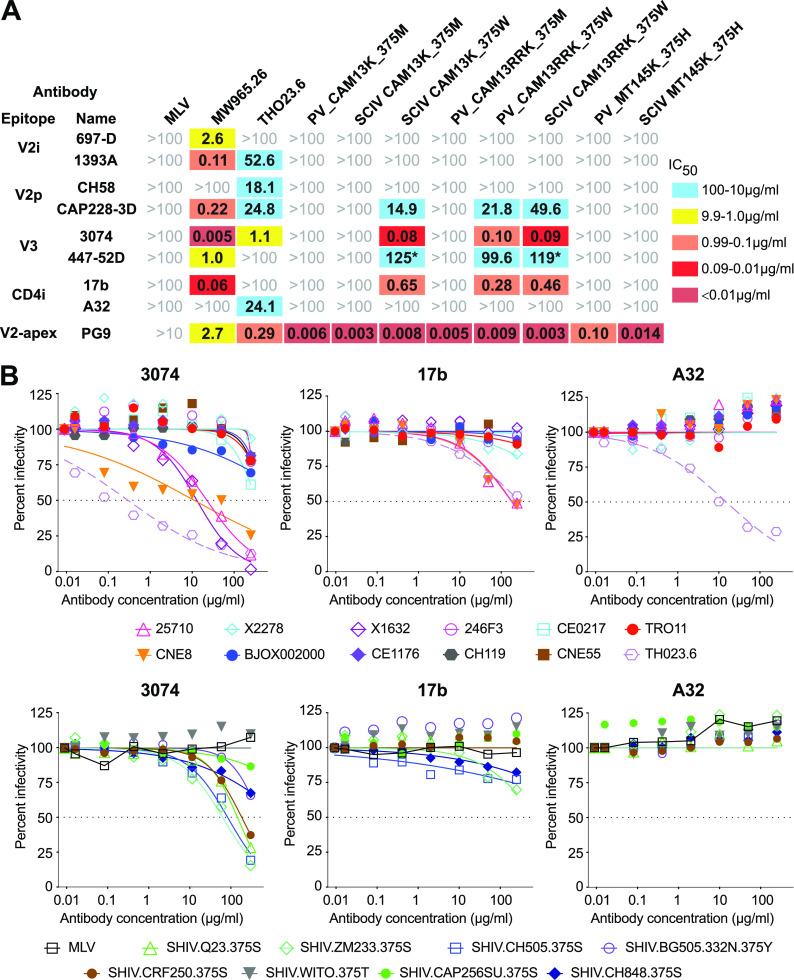
SCIV expressed, but not wild-type SIVcpz Env, as well as some primary HIV-1 Envs adopting a more open conformation. (A) The sensitivity of CAM13K and CAM13RRK Envs to nonneutralizing V2p, V2i, and linear V3 and CD4i antibodies as well as the V2-apex bNAb PG9 (indicated on the left) is shown for pseudovirus (PV) and SCIV constructs encoding either the wild-type methionine (M) or the RM-selected tryptophan (W) at position 375 (indicated on top). Fifty percent inhibitory concentrations (IC_50_) are shown in μg/mL (coloring indicates relative neutralization potency, with asterisks identifying IC_50_ values estimated by the Prism software). Also shown are IC_50_ values for pseudovirus and SCIV-expressed MT145K Env, which encodes a histidine at position 375. MLV and tier 1 HIV-1 (MW965.26, TH023.6) Env pseudoviruses are shown for control. (B) Neutralization curves are shown for nonneutralizing V3 (3074) and CD4i (17b, A32) antibodies (indicated on top) against global panel pseudoviruses (upper panel) and SHIV strains (lower panel; with the amino acid residue at position 375 indicated). Dashed lines indicate 50% reduction in virus infectivity.

To determine whether the CAM13K and CAM13RRK Envs inherently adopt a more open conformation, we tested their sensitivity to conformation-sensitive V2i (697-D, 1393A) (62), V2p (CH58, CAP228-3D) (42, 63, 64), linear V3 (3074, 447-52D) (65, 66), and CD4i (17b, A32) (67, 68) antibodies as well as the V2-apex bNAb PG9 for control. These antibodies were selected because they recognize epitopes that are occluded in prefusion trimers and thus only neutralize viruses whose Envs are incompletely closed. Using antibodies up to a concentration of 100 μg/mL, we found that pseudoviruses (PV) carrying full-length CAM13K and CAM13RRK Envs with a wild-type methionine at position 375 were completely resistant to the conformation-sensitive V2, V3, and CD4i antibodies (Fig. 7A). This was also observed for a SCIV construct with a methionine at position 375, thus excluding the possibility that the chimeric gp41 domain influenced the stability of the timer apex. In contrast, *in vivo* selected CAM13K and CAM13RRK SCIV constructs with a tryptophan at position 375, as well as a CAM13RRK pseudovirus in which the 375M was mutated to a 375W, were all sensitive to monoclonal antibodies CAP228-3D, 3074, 447-52D, as well as 17b (Fig. 7A). These results indicate that the amino acid substitution at position 375 rendered CAM13K and CAM13RRK Env conformationally more flexible, with the bulky 375W shifting the equilibrium toward a more open state. Interestingly, the SIVcpz MT145K Env, which naturally encodes a histidine at position 375, was resistant to neutralization by CAP228-3D, 3074, 447-52D, and 17b, both as a pseudovirus and as a SCIV construct (Fig. 7A). Thus, the propensity to adopt a more open conformation is not an inherent property of SIVcpz Envs or SCIV constructs, but is an Env-specific characteristic.

Finally, we examined the conformational state of global panel viruses and SHIV strains that were used to characterize the SCIV-induced cross-neutralizing antibodies (Fig. 7B). As reported previously (52), we found that several global panel pseudoviruses were sensitive to the linear V3 antibody 3074. This was also true for SHIV.Q23, SHIV.ZM233, SHIV.CRF250, and SHIV.CH505, which despite encoding a wild-type amino acid residue at position 375 were sensitive to 3074 neutralization, but only at high antibody concentrations (IC_50_ >50 μg/mL). Although none of the tier 2 viruses were neutralized by A32, global panel strains 25710 and CNE8 were sensitive to 17b, as was SHIV.ZM233, although the latter virus did not reach an IC_50_ (Fig. 7B). Thus, some primary HIV-1 Envs are prone to sample a more open conformation and to expose a cryptic V2 epitope in the context of an occluded-open trimer. Although the V1V2 region is highly variable, several sites in the C strand are relatively conserved, thus explaining the sensitivity of these HIV-1 strains to neutralization by SIVcpz Env-induced antibodies.

## DISCUSSION

The HIV-1 trimer apex elicits both nonneutralizing and neutralizing antibodies, including some of the most potent bNAbs identified to date (69). Here, we analyzed the antigenic conservation of the V2-apex across divergent SIV lineages. We found that SIVs infecting African apes and some Old World monkeys are exquisitely sensitive to neutralization by mature human V2-apex bNAbs, indicating that the quaternary structure of the trimer apex is highly conserved. This sensitivity was further increased by modifications in the C strand, which also rendered two new SIVcpz Envs susceptible to neutralization by inferred human V2-apex bNAb precursors. Thus, SIV Envs, and especially SIVcpz Envs, may have utility as germ line-targeting and/or immunofocusing components of an AIDS vaccine. While priming with an SIV trimer would generate B cell responses to the entire Env surface, a subsequent boost with an HIV-1 trimer that shared only the V2-apex bNAb site should focus the B cell recall response to this epitope. In addition, the backbone diversity of the SIV Envs may reduce germinal center competition for B cells with long HCDR3s that target the V2-apex. There is increasing evidence that sequential immunization with divergent HIV-1 Env immunogens favors B cell recall responses to shared epitopes (22 to 24, 26, 70). Thus, the additional SIV Envs characterized here provide a unique collection to develop a wider array of priming and boosting immunogens.

To test the utility of an SIVcpz Env priming immunogen, we generated a soluble CAM13K SOSIP trimer and evaluated its germ line-targeting properties in knock-in mice that expressed a precursor of the human V2-apex bNAb CH01 (20). We found that CAM13K SOSIP immunization elicited antibodies that neutralized CH01_RUA-sensitive HIV-1 strains in an N160-dependent manner, demonstrating the stimulation and expansion of precursor antibody-expressing mouse B cells. However, this knock-in mouse model expresses only a single prearranged inferred germ line heavy chain at an unphysiologically high precursor frequency. Moreover, the RUA of CH01, like that of PG9 or PG16, was inferred from very few lineage members and thus represents only an approximation of the unmutated common ancestor (35, 69). Given these limitations, it seems clear that the germ line-targeting potential of the CAM13K Env and future derivatives will have to be confirmed in additional mouse models, such as long human CDR3 rearranging mice, as well as ultimately in outbred animals and humans (71 to 73).

We recently reported that SHIVs expressing transmitted founder Envs, which in humans elicited bNAbs, did the same in RMs, with the molecular patterns of Env-Ab coevolution mirroring those observed in HIV-1-infected humans (12). Using a similar approach to examine the bNAb induction potential of SIVcpz Envs, we thus cloned CAM13K and CAM13RRK Envs into the same SHIV vector and used the resulting SCIV constructs to infect RMs. We found that the two SCIVs closely resembled SHIVs in their *in vivo* replication kinetics and other key biological properties. Both SCIVs caused persistent infections *in vivo*, resulting in high peak (10^6^ to 10^8^ copies/mL) and setpoint (10^3^ to 10^5^ copies/mL) viral loads. Both SCIVs preferred a bulky aromatic amino acid at position 375, which has been shown to confer enhanced affinity to the rhesus CD4 receptor and thus an improved ability to replicate *in vivo* (21, 74). Finally, both SCIVs induced potent autologous tier 2 neutralizing antibodies, consistent with a high antigenic burden and the expression of intact Env trimers. Recent data have shown that SHIV infections are not only useful for tracing bNAb development, but also represent an important outbred animal model for HIV-1 transmission, prevention, immunopathogenesis, and cure studies (75 to 82). Our data show that this model is not restricted to HIV-1 Envs but can be extended to more divergent Envs from primate lentiviruses.

Given that a subset of SHIV-infected RMs develop V2-apex bNAbs that closely resemble those of humans (12), we reasoned that SCIV infection may induce similar responses. Indeed, six of seven RMs developed limited plasma breadth that mapped to the C strand and was accompanied by rapid viral escape at positions 169 and 186 in the V2 loop, suggesting nascent bNAb development (12). However, neither SCIV.CAM13K- nor SCIV.CAM13RRK-infected RMs ultimately developed V2-apex bNAbs, although B cell cloning identified antibodies that cross-neutralized a number of tier 2 HIV-1 strains with low potency. Negative stain electron microscopy, coupled with conformational probing of SCIV and wild-type SIVcpz Envs, provided an explanation: the introduction of a tryptophan at position 375, which mediated efficient *in vivo* replication, rendered SCIV-expressed CAM13K and CAM13RRK Envs conformationally more open, as exemplified by an increased sensitivity to V2p, linear V3, and CD4i antibodies. This propensity likely impeded the development of bNAbs, even if appropriate V2-apex bNAb precursors were stimulated, as suggested by the expansion of long HCDR3 antibody lineages, including many that encoded the HD3-9*01 gene thus far identified in all RM-derived V2-apex bNAbs.

The finding of a cryptic V2 epitope that is exposed on occluded-open, but not closed, Env trimers is consistent with recent structural studies of antibodies detected in sequentially SOSIP-immunized RMs (28). Examining immunization-elicited neutralizing antibodies that target the CD4bs, Yang and colleagues found that Fab binding was only observed when Envs were not constrained in their closed conformation (28). Instead, these CD4bs bNAbs bound an occluded-open trimer, which also exposed V1V2 regions that were buried in closed trimers. Although no antibody specific for these regions was isolated, the cryo-EM data suggested the presence of a cryptic epitope (28). Our results confirm and extend these findings by showing that cryptic V2 epitopes are present not only on SOSIP trimers but also on membrane-anchored Envs, which has implications for mRNA immunogen design.

In addition to prototypic V2-apex bNAbs, two classes of V2-directed nonneutralizing antibodies have been described. One of these, termed V2p, includes both human and macaque antibodies that recognize the V2 loop in a helix-coil conformation and require a glutamic acid-aspartic acid (ED) LCDR2 motif for V2 peptide binding (42, 63, 64). The other class, designated V2i, recognizes conformational epitopes on gp120 (83) and has recently been shown to belong to the large family of CD4i antibodies (61). Our SCIV-derived antibodies lack the ED LCDR2 motif and bind the V2 domain in an occluded-open trimer. Moreover, they neutralize primary viruses, whereas V2p and V2i antibodies only neutralize tier 1 isolates (42, 63, 84). Thus, the SCIV-induced antibodies make up a new, fourth class of V2-directed antibodies, which we term V2w for their weakly cross-neutralizing activity.

Although the SCIV-induced cross-neutralizing antibodies represent off-target responses, these findings do not indicate that SIVcpz Envs are unsuitable immunogens. CAM13K and CAM13RRK Envs with a methionine at position 375 were resistant to V2, linear V3, and CD4i antibodies, indicating a closed conformation, and this was also true for the SIVcpz MT145K Env, both in the context of pseudovirus and SCIV constructs. Moreover, SHIV-expressed Envs are not inherently more open than the corresponding wild-type Envs, as recently shown for 10 transmitted founder Env-expressing SHIVs (21). Nonetheless, it is clear that the HIV-1 envelope glycoprotein is conformationally flexible (85, 86), and that some HIV-1 strains sample open Env conformations more readily than others (52, 87). Since the latter are likely to elicit neutralizing responses similar to the ones described here, it will be necessary to use conformation-sensitive probes to examine immunogens and SHIV/SCIV constructs prior to vaccination and infection studies. On the other hand, greater conformational flexibility may also have benefits, e.g., by enhancing Env binding to bNAb precursors. It will thus be important to explore both the advantages and disadvantages of Env immunogens that reversibly expose some surfaces that are buried in closed Env trimers.

In summary, we examined the antibody response to germ line-targeting versions of the SIVcpz CAM13 Env, both as a soluble SOSIP immunogen and as infectious SCIVs in RMs. Although SCIV infection failed to elicit canonical V2-apex bNAbs, we found that C-strand modified versions of the CAM13 Env bound several human V2-apex bNAb precursors and stimulated one of these in knock-in mice. We also characterized a new class of V2-directed antibodies that targets a conserved epitope in occluded-open SIVcpz and HIV-1 Env trimers. Although the breadth and potency of this new specificity are limited, our findings expand the spectrum of V2-apex targeted antibodies that can contribute to neutralization breadth and identify a novel SIVcpz Env platform for further development as germ line-targeting and immunofocusing immunogens.

## MATERIALS AND METHODS

### Animal studies.

CH01_RUA homozygous “HC only” (V_H_DJ_H_^+/+^) knock-in (KI) mice were housed at Duke University and cared for in an Association for Assessment and Accreditation of Laboratory Animal Care (AAALAC)-accredited facility (20). All animal procedures were approved by the Duke Institutional Animal Care and Use Committee (IACUC). Five mice received 20 μg of the CAM13K SOSIP trimer plus 5 μg of GLA-SE adjuvant, while three mice received 5 μg of GLA-SE alone. All animals were immunized six times at 2-week intervals by intramuscular injection at two sites (25 μL each) in the hind limbs (Fig. 2A). Blood samples were collected at prebleed (pre) and 1 week after the last immunization, and sera were tested for neutralizing activity using autologous and CH01_RUA-sensitive heterologous pseudoviruses. Serum samples were heat inactivated for potential complement activity at 56°C for 30 min.

Indian rhesus macaques were housed at Bioqual, Inc., Rockville, Maryland, according to guidelines of the AAALAC standards. Experiments were approved by the University of Pennsylvania and Bioqual Institutional Animal Care and Use Committees (IACUC). All RMs were socially housed with a variety of recommended environmental enrichments and sedated for blood draws, anti-CD8 MAb infusions, and SCIV inoculations. All animals received an intravenous infusion of 25 mg/kg of the anti-CD8b MAb CD8beta255R1 at the time of SCIV inoculation. RMs T925, T926, and T927 received 1 mL of a SCIV.CAM13K infection stock containing six variants differing at Env position 375 (50 ng of p27 antigen each) by intravenous injection. RMs T281, T282, V032, and 12D010 received 1 mL of SCIV.CAM13RRK, which encoded a W at position 375 (50 ng of p27 antigen). Animals underwent sequential blood draws to obtain plasma and PBMCs, and lymph node cells were obtained at necropsy. Plasma viral loads were determined as previously described (12, 21). Three SCIV.CAM13RRK-infected animals were repurposed from prior HIV-1 immunization studies. T281 and T282 received HIV-1 subtype C peptides containing liposomes, while V032 received CH505 SOSIP immunizations as described (88). Although preinfection plasmas of these animals weakly neutralized the tier 1 strain MW965.26, none had detectable tier 2 neutralizing antibodies (Fig. 3D). Animal 12D010 developed progressive pancytopenia.

### SIV Env expression plasmids.

The nucleotide sequences of the SIV Envs used for V2-apex neutralization have been reported (see references and GenBank accession numbers in Table S2A at https://doi.org/10.6084/m9.figshare.21644675), except for the CPZ.Pts.TAN10 Env, which was amplified from the spleen of a naturally SIVcpz-infected chimpanzee (Ch-036) who died of an AIDS-like illness in Gombe National Park (89), and the CPZ.Pts.ANT_Cot Env, which was derived from a limiting dilution plasma viral isolate obtained from an SIVcpzANT-infected captive chimpanzee (90). SIV Env pseudotypes were produced in 293T cells by cotransfection with an Env-deficient SIVcpzMB897-EnvFS backbone (see GenBank accession numbers in Table S2A at https://doi.org/10.6084/m9.figshare.21644675), titered (19), and tested for sensitivity to the mature V2-apex bNAbs and their inferred precursors in the TZM-bl assay (see Table S2B at https://doi.org/10.6084/m9.figshare.21644675 for GenBank accession numbers of heavy and light chain variable domain sequences of all antibodies used). A subset of SIV Envs was further modified by site-directed mutagenesis to improve germ line-targeting (see Table S2C at https://doi.org/10.6084/m9.figshare.21644675 for GenBank accession numbers).

### Neutralizing antibody assay.

The neutralization capacity of rhesus macaque plasma and monoclonal antibodies was assessed using the TZM-bl assay as described (39). Briefly, serial 5-fold dilutions of RM plasma (1:20, 1;100, 1:2,500, 1:12,500, 1:62,500, 1:312,500) or monoclonal antibodies (e.g., 250, 50, 10, 2, 0.04, 0.08, 0.016, 0.00 μg/mL) were incubated with transfection-derived virus at a multiplicity of infection of 0.3 in a total volume of 100 μL in the presence of DEAE-dextran (40 μg/mL) for 1 h at 37°C, and this mixture was then added to TZM-bl cells. After 48 h, TZM-bl cells were analyzed for luciferase expression, with uninfected cells used to correct for background luciferase activity. The infectivity of each virus without plasma or antibodies was set at 100%, and the plasma dilution or antibody concentration that reduced the relative light units (RLUs) by 50% compared with the no Ab control wells were calculated by using the variable slope (four parameters) function in Prism software (v8.0).

Viral stocks were generated by transfection of 293T cells. Briefly, cells were transfected by adding 0.5 mL of a preincubated DMEM solution containing 4.5 μg of SIVcpz (SIVcpzMB897-EnvFS) or HIV-1 (SG3Δenv) backbone plasmids and 30 ng of codon-optimized or 1.5 μg of wild-type SIVcpz and HIV-1 Env plasmids, respectively, or 6 μg of SCIV or SHIV construct DNA, and 18 μL of FuGENE 6 transfection reagent (Promega). The cells were incubated at 37°C for 48 to 72 h, and supernatant was harvested and stored at −80°C.

### SCIV construction.

SCIV.CAM13K was generated using a second-generation SHIV vector (pCRXTOPO.SHIV.V2.backbone1) as described (21). This vector allowed the unidirectional cloning of a CAM13K *vpu-env* fragment (*env* nucleotides 1 to 2153, HXB2 numbering) into unique BsmBI restriction enzyme sites. The CAM13K *vpu-env* fragment was amplified from the SIVcpz CAM13 proviral clone (43), followed by a glutamine-to-lysine mutation at position 171 (Q171K) to improve V2-apex bNAb germ line-targeting (20). The SCIV construct was then used to create allelic variants at position 375, and each SCIV genome was sequence confirmed. The SCIV.CAM13RRK construct was generated by replacing two wild-type lysine residues at positions 169 and 170 in the Env of SCIV.CAM13K_375W with arginine residues (see Table S2A at https://doi.org/10.6084/m9.figshare.21644675 for GenBank accession numbers of SCIV.CAM13K and SCIV.CAM13RRK).

### Single genome amplification.

Briefly, ~20,000 copies of viral RNA were extracted from plasma using the QIAamp Viral RNA kit (Qiagen) and reverse transcribed using SuperScript III Reverse Transcriptase (Invitrogen). Viral cDNA was then endpoint diluted, and 3′ half genomes or viral *env* genes were amplified using nested PCR with primers and conditions as previously reported (91, 92). Geneious software was used for alignments and sequence analysis (see Table S2D at https://doi.org/10.6084/m9.figshare.21644675 for GenBank accession numbers of longitudinal *env* gene sequences).

### Longitudinal Env evolution in SCIV-infected RMs.

Longitudinal Env evolution analyses were performed as described (12). Briefly, LASSIE (https://github.com/phraber/lassie) was used to identify amino acid/glycan mutations under selection, using 80% or higher loss of the transmitted virus sequence as the cutoff. The webtool AnalyzeAlign was used to calculate sequence logos (https://www.hiv.lanl.gov/content/sequence/ANALYZEALIGN/analyze_align.html) that show evolution at such sites. Glycan shield mapping was performed using the Glycan Shield Mapping tool (https://www.hiv.lanl.gov/content/sequence/GLYSHIELDMAP/glyshieldmap.html) (56). Hypervariable loops from longitudinal Envs were characterized using alignment-free characteristics (https://www.hiv.lanl.gov/content/sequence/VAR_REG_CHAR/index.html), such as length, number of glycans, and net charge. Hypervariable loop positions were identified based on HXB2 numbering:132 to 152 for hypervariable V1,185 to 190 for hypervariable V2, 396 to 410 for hypervariable V4, and 460 to 465 for hypervariable V5.

### Biotinylated trimer probes for B cell sorting.

AVI-tagged ZM197-ZM233V1V2 (20) and CAP256-wk34c80-RnS-3mut-2G (55) SOSIP trimers were produced in FreeStyle 293F cells (ThermoFisher Scientific) by premixing C-terminal AVI-tagged ZM197-ZM233V1V2 or C-terminal AVI-tagged CAP256-wk34c80-RnS-3mut-2G with a furin expression plasmid using Polyethylenimine (PEI) 40K (Polysciences, Inc.) or Turbo293 (Speed BioSystems) transfection reagents, respectively. The cells were cultured for 4 to 6 days before AVI-tagged trimers were purified from supernatants using either PGT145 or 2G12 affinity chromatography. Protein A resin-captured AVI-tagged ZM197-ZM233V1V2 was biotinylated with BirA enzyme (Avidity), cleaved from the Fc purification tag with HRV3C protease, concentrated using Amicon Ultra-15-30K (Millipore), and purified using a Superdex 200 16/600 gel filtration column (Cytiva). The 2G12 affinity column captured AVI-tagged CAP256-wk34c80-RnS-3mut-2G protein was eluted with 3 M magnesium chloride solution, concentrated using Centricon Plus-70-30K (Millipore), followed by a Superdex 200 16/600 gel filtration column. The corresponding trimer fractions were pooled, negatively selected using a V3 cocktail column containing six V3-directed antibodies (1006-15D, 2219, 2557, 2558, 3074, and 50.1), and biotinylated with BirA enzyme followed by final purification over a Superdex 200 16/600 gel filtration column.

### B cell sorting.

Single HIV-1 Env–specific B cells were isolated from the blood (weeks 24 and 32) and lymph node (week 62) of RM T927 and from the blood (week 24) of RM T925, both of which were infected with SCIV.CAM13K. Cryopreserved cells were thawed, stained with LIVE/DEAD Fixable Aqua Dead Cell Stain (Life Technologies), and washed and stained with an antibody cocktail against CD3 (clone SP34-2, BD Biosciences), CD4 (clone OKT4, BioLegend), CD8 (clone RPA-T8, BioLegend), CD14 (clone M5E2, BioLegend), CD20 (clone 2H7, BioLegend), IgG (clone G18-145, BD Biosciences), IgD (polyclonal, Dako), and IgM (clone G20-127, BD Biosciences) at room temperature in the dark for 30 min. Cells were washed and incubated with AVI-tagged and biotinylated ZM197-ZM233V1V2 (20) and CAP256-wk34c80-RnS-3mut-2G (55) SOSIP trimers conjugated to phycoerythrin (PE) or allophycocyanin (APC) for 30 min at room temperature. The stained cells were then washed three times with PBS, resuspended in 1 mL of PBS containing 5% FBS and 0.1% sodium azide, and passed through a 70-μm cell mesh (BD Biosciences). Since double positive cells were rare, memory B cells that bound to one or the other of these probes (CD3-CD4-CD8-CD14-CD20+IgD-IgM-IgG+probe+) were isolated with a FACSAria cell sorter using the FACSDiva software (BD Biosciences), and flow cytometric data were subsequently analyzed using FlowJo (v10.8.1). B cells were sorted at 1 cell per well in a 96-well plate containing 20 μL lysis buffer as described (12). Plates were frozen on dry ice and stored at −80°C.

### Rhesus macaque monoclonal antibody production.

Heavy and light chain variable region genes were amplified using single-cell PCR approaches as described (93). Briefly, immunoglobulin gene transcripts from single B cells were reverse transcribed with Superscript III (Invitrogen) using random hexamer primers (Thermo Scientific). The cDNA was then used as a template for two rounds of nested PCR for heavy and light chain amplification, with amplicons examined by agarose gel electrophoresis. Amplicons were Sanger sequenced (Genewiz), and gene sequences were computationally analyzed using SONAR (94), IgBLAST (95), and IMGT V-QUEST (96). SONAR provided initial gene assignment, sequence annotation, and clonotype clustering based on CDR3 similarity, while IgBLAST and V-QUEST provided additional annotations. For V-QUEST, the IMGT rhesus macaque reference database was used, while for SONAR and IgBLAST an alternate rhesus gene reference database with additional diversity was used (58). Final lineage assignments were made after review of all automatic annotations and grouping results by looking for evidence of common descent, such as similar junctions and shared somatic hypermutation. As a final step of germ line gene assignment, all heavy chain sequences were compared to a recent macaque reference (57), and the closest-matching gene was retained as the final germ line gene.

To produce select antibodies, paired heavy (VDJ) and light (VJ) chain variable gene sequences were commercially synthesized (GenScript) and cloned into antibody expression plasmids (see Table S2E at https://doi.org/10.6084/m9.figshare.21644675 for GenBank accession numbers of heavy and light chain variable regions). Recombinant antibodies were produced by cotransfecting paired heavy and light chain expression plasmids into Expi293F cells using ExpiFectamine 293 transfection reagents, purified from culture supernatants using the Protein A/Protein G GraviTrap kit, and buffer-exchanged into PBS as described (12).

Fab fragments were generated by inserting a stop codon six amino acids upstream of the hinge region (CPPCP) of the heavy chain expression plasmid, which was then cotransfected with the respective light chain plasmid, harvested by centrifugation, and then passed through a CaptureSelect CH1-XL Affinity Matrix column (ThermoFisher Scientific). Recombinant Fab protein was eluted with 50 mM acetate buffer at pH 5.0 and concentrated using Ultra-15 Centrifugal Filters (Millipore). Protein concentration was determined using the Qubit protein assay (Invitrogen).

### Biolayer interferometry.

The antigenicity of the CAM13K SOSIP trimer was examined by biolayer interferometry (BLI) using an OctetRed96 platform (Sartorius) and a panel of monoclonal antibodies as shown in Fig. S1. Antibodies were diluted to 20 μg/mL and captured onto anti-hIgG Fc capture (AHC) biosensors for a period of 300 s and then washed with PBS pH 7.4. The antibody captured biosensors were then submerged into wells containing CAM13K SOSIP protein diluted to 50 μg/mL for 400 s followed by a dissociation period of 600 s in 1× PBS. Nonspecific binding was assessed using an anti-influenza hemagglutinin MAb (CH65) for reference subtraction, and the binding curves were analyzed using the ForteBio Data Analysis Software 10.0 (Sartorius). Binding responses (nm) of the SOSIP to each antibody were measured at 390 to 395 s of the association phase after reference subtraction, and the binding response was normalized relative to PGT145.

### CAM13K and CH505.N156Q SOSIP trimer expression.

To generate a soluble CAM13K Env trimer immunogen, we introduced several stabilizing mutations as shown in Fig. S1A. Similarly, to generate a CH505.N156Q SOSIP trimer, we mutagenized the CH505 transmitted founder Env and stabilized it with a chimeric gp41 as well as E64K and A316W substitutions to reduce V3 loop exposure (97). SOSIP expression plasmids were transfected into Freestyle 293F cells together with a furin-encoding plasmid to improve gp120/gp41 cleavage. Six days posttransfection, the supernatant was cleared of cells, concentrated, filtered, and subjected to PGT145 affinity chromatography by gravity flow columns or on a AKTA Pure (Cytvia) as previously described (97). Protein eluted from the PGT145 affinity column was 0.2 μm-filtered and concentrated prior to performing size exclusion chromatography. SOSIP trimers were purified in 10 mM Tris pH 8, 500 mM NaCl on a HiLoad Superose6 16/600 column (Cytvia).

### Negative stain electron microscopy.

Fab-SOSIP complexes were formed by mixing 10 μg of SOSIP with 36 μg of Fab, and bringing the final volume to 100 μL with buffer containing 20 mM HEPES, 150 mM Na_2_SO_4_, and 5 mM NaN_3_, pH 7.4, and incubating overnight at 22°C. The next day, samples were diluted with 900 μL of buffer, then 1 mL of 0.16% glutaraldehyde in the same buffer was added and incubated for 5 min. We added 160 μL of 1 M Tris buffer, pH 7.4, to quench unreacted glutaraldehyde, and samples were transferred to a 2-mL, 100-kDa Amicon centrifugal concentrator and spin-concentrated to ~50 μL. Concentrated samples were then diluted to a nominal SOSIP concentration of 0.2 mg/mL with buffer containing 20 mM HEPES, 150 mM NaCl, and 5 g/dL glycerol, applied to a glow-discharged carbon-coated EM grid for 8 to 10 s, blotted and stained with 2 g/dL uranyl formate for 1 min, and then blotted and air-dried. Grids were examined on a Philips EM420 electron microscope operating at 120 kV and nominal magnification of 49,000×, and ~100 images for each sample were collected on a 76 Mpix CCD camera at 2.4 Å/pixel. Images were analyzed by 2D class averages and 3D reconstructions calculated using standard protocols with Relion 3.0 (98).

### Statistical analyses.

Differences between immunized and adjuvant-only knock-in mouse groups were assessed using a nonparametric Mann-Whitney test using GraphPad Prism 9.4.0 software. We chose a nonparametric rank-based test because the distributions of antibody titers can be highly variable and we had small sample sizes. Statistical significance of hypervariable loop characteristics was determined using a nonparametric two-sided Kendall Tau test.

### Data availability.

All GenBank accession numbers are listed in Table S2 at https://doi.org/10.6084/m9.figshare.21644675.
