# Supplementary material for: A Germline-Targeting Chimpanzee SIV Envelope Glycoprotein Elicits a New Class of V2-Apex Directed Cross-Neutralizing Antibodies
Source: mBio. 2023 Jan 11;14(1):e03370-22. doi: 10.1128/mbio.03370-22 (PMC9973348; doi:10.1128/mbio.03370-22)
Supplement: TABLE S1 [file mbio.03370-22-s0010.pdf]

Table S1. Neutralization sensitivity of wildtype and modified SIV Envs to V2-apex bNAbs and their inferred precursors

Neutralization sensitivity[1]	PG9 RUA[2]	PG16 RUA[2]	VRC26 UCA[2]	CH01 RUA[2]	PG9	PG16	VRC26.25	CH01
SIVcpz.LB7	>50	>50	>50	>50	>10	1.4	0.08	>10
SIVcpz.LB7 Q171K	>50	>50	>50	>50	0.73	>10	0.07	7.6
SIVcpz.MB897	>50	>50	>50	>50	0.09	0.05	0.001	>10
SIVcpz.MB897 Q171K	>50	>50	>50	>50	0.03	0.01	0.001	1.2
SIVcpz.MB897 Q171K + N130K	>50	>50	>50	>50	0.003	0.003	0.0007	0.18
SIVcpz.MB66	>50	>50	>50	>50	>10	0.01	0.07	>10
SIVcpz.MB66 Q171K	>50	>50	>50	>50	>10	5.8	0.04	>10
SIVcpz.CAM13	>50	>50	>50	>50	0.02	0.09	0.002	>10
SIVcpz.CAM13 Q171K	28.2	37.7	>50	>50	0.007	0.003	0.002	0.09
SIVcpz.MT145	>50	>50	>50	>50	0.16	0.34	0.0003	>10
SIVcpz.MT145 Q171K	>50	>50	>50	2.7	0.04	0.03	0.002	0.34
SIVcpz.DP943.2	>50	>50	>50	>50	0.02	0.02	0.001	6.8
SIVcpz.DP943.2 Q171K	>50	>50	>50	1.0	0.007	0.006	0.0007	0.06
SIVcpz.DP943.2 Q171K + N156 glycan (K156N+F158S)	>50	>50	>50	49.1	0.01	0.009	0.009	0.15
SIVgor.BPID1	>50	>50	>50	>50	>10	>10	>10	>10
SIVgor.BPID1 E170K + Q171K	>50	>50	>50	>50	>10	4.3	>10	>10
SIVgor.CP2139	>50	>50	>50	>50	>10	>10	>10	>10
SIVgor.CP2139 T169K + T170K	>50	>50	>50	>50	0.12	0.76	>10	2.8
SIVgor.CP2139 T169K + T170K + K166R	>50	>50	>50	>50	0.14	8.1	0.008	>10
SIVgor.CP2139 T169K + T170K + K166R + N130K	>50	>50	>50	>50	0.01	9.6	0.004	>10
SIVgor.BQID2	>50	>50	>50	>50	>10	4.9	>10	>10
SIVgor.BQID2 S170K + Q171K	>50	>50	>50	>50	0.45	0.79	>10	>10
SIVgor.BQID2 S170K + Q171K + N130T	>50	>50	>50	>50	3.2	>10	>10	>10
SIVcpz.TAN10	>50	>50	>50	>50	>10	1.9	>10	5.6
SIVcpz.TAN10 Q171K	>50	>50	>50	>50	0.13	>10	>10	7.0
SIVmus.11GAB	n.t	n.t	n.t	n.t.	0.54	9.5	0.54	>10
SIVmus.11GAB Q171K	>50	>50	>50	>50	0.12	1.8	0.92	6.5
SIVmus.1085 4-12	n.t	n.t	n.t	n.t.	0.14	0.006	0.03	>10
SIVmus.1085 4-12 Q171K	>50	>50	>50	>50	0.03	0.04	0.02	1.17
SIVasc.RT11	n.t	n.t	n.t	n.t.	0.47	>10	>10	>10
SIVasc.RT11 H171K	>50	>50	>50	>50	0.09	>10	>10	9.3
SIVasc.RT11 H171K + K166R	>50	>50	>50	>50	0.05	0.55	>10	2.6
SIVasc.RT11 H171K + A170K	>50	>50	>50	>50	0.31	>10	>10	>10

[1]Numbers indicating IC_{50} values (μg/ml) as determined in the TZM-bl assay (n.t., not tested). The highest antibody concentration used was 50 μg/ml (colors indicate relative neutralization potency).
[2]RUA, reverted unmutated ancestor; UCA, unmutated common ancestor.
