# Supplementary material for: A Germline-Targeting Chimpanzee SIV Envelope Glycoprotein Elicits a New Class of V2-Apex Directed Cross-Neutralizing Antibodies
Source: mBio. 2023 Jan 11;14(1):e03370-22. doi: 10.1128/mbio.03370-22 (PMC9973348; doi:10.1128/mbio.03370-22)
Supplement: FIG S1 [file mbio.03370-22-s0001.pdf]

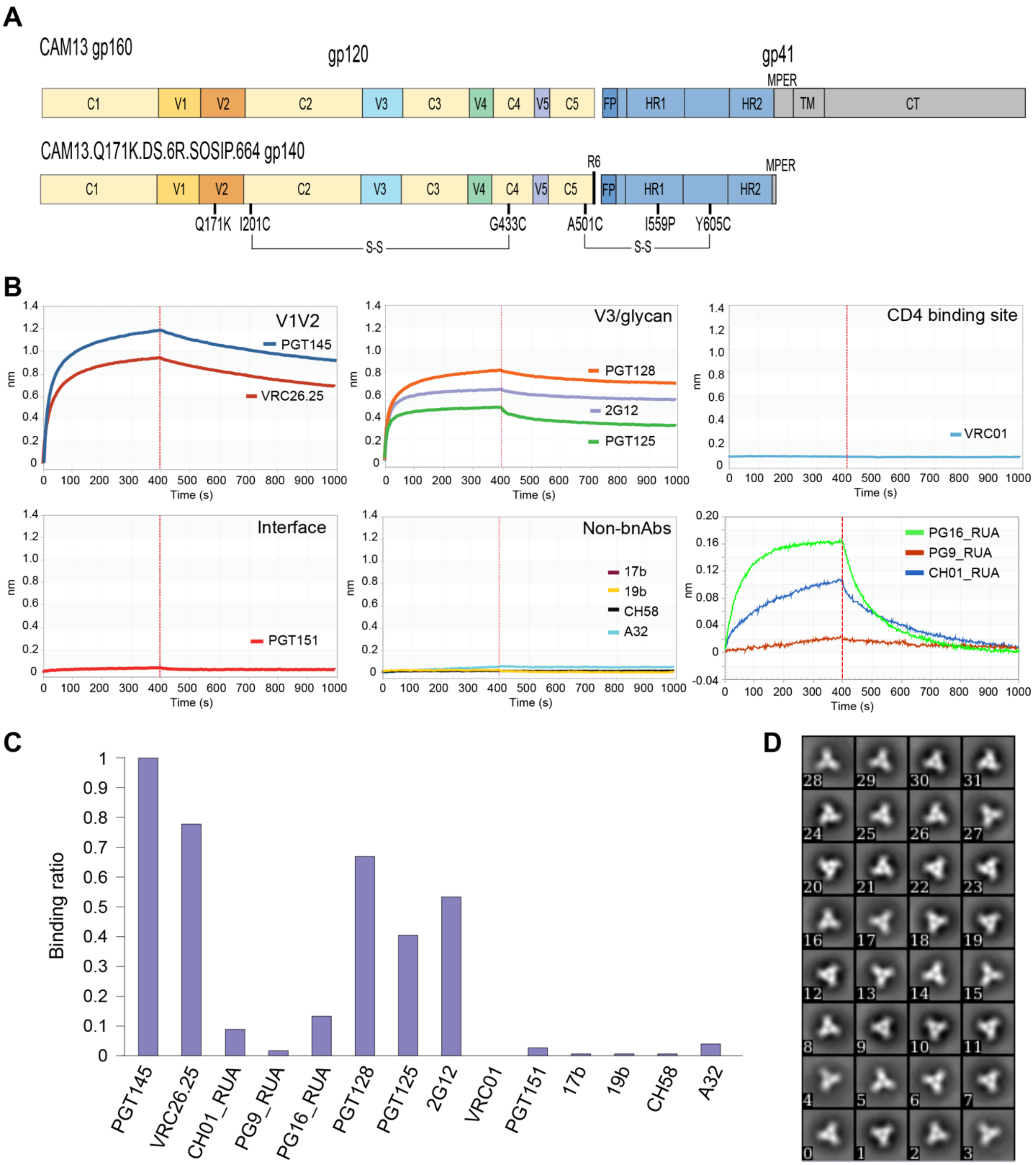

FIG S1 Generation and characterization of a CAM13K SOSIP trimer. (A) The CAM13K SOSIP construction scheme is shown in relation to the wildtype SIVcpz CAM13 gp160, with the location of constant (C) and variable (V) gp120 segments as well as functional gp41 domains (FP, fusion

peptide; HR1, heptad repeat 1; HR2, heptad repeat 2, MPER, membrane proximal external region; TM, transmembrane domain; CT, cytoplasmic domain) indicated. In addition to the Q171K mutation, the CAM13K SOSIP trimer was designed to include the original SOSIP.664 mutations (A501C-Y605C; I559P; truncation at residue 664) (45, 46), the DS stabilizing mutation (I201C-G433C) (47), as well as a hexa-arginine furin cleavage site (R6) (48). (B). Biolayer interferometry (BLI) sensorgrams depicting the binding of the CAM13K SOSIP trimer (at 50 μg/ml) to anti-human IgG Fc captured mature V2-apex (PGT145, VRC26.25), V3-glycan (PGT128, 2G12, PGT125), CD4 binding site (VRC01) and interface (PGT151) bNAbs as well as non-neutralizing V2 (CH58), V3 (19b), and CD4-induced (17b, A32) antibodies. Sensorgrams depicting the binding of the V2-apex bNAb precursors PG16_RUA, PG9_RUA, and CH01_RUA are also shown. (C) Antibody binding of the CAM13K SOSIP trimer relative to PGT145. Binding ratios were calculated relative to the binding of PGT145 which showed the highest level of binding (ratio = binding to mAb [nm] / binding to PGT145 [nm]). (D) Two-dimensional class averages of NSEM images of the CAM13K SOSIP protein showing the expected trimer morphology.
