# Supplementary material for: A Germline-Targeting Chimpanzee SIV Envelope Glycoprotein Elicits a New Class of V2-Apex Directed Cross-Neutralizing Antibodies
Source: mBio. 2023 Jan 11;14(1):e03370-22. doi: 10.1128/mbio.03370-22 (PMC9973348; doi:10.1128/mbio.03370-22)
Supplement: FIG S2 [file mbio.03370-22-s0002.pdf]

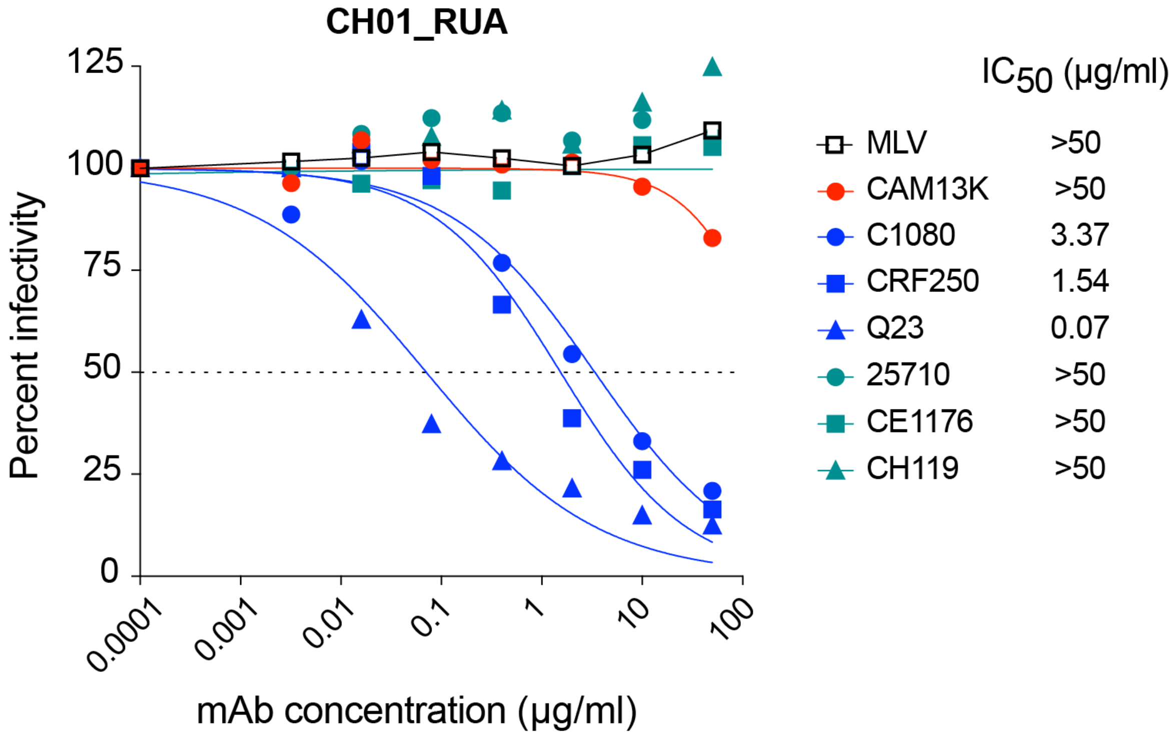

FIG S2 Sensitivity of HIV-1 Env pseudoviruses to neutralization by CH01_RUA. Pseudoviruses carrying different HIV-1 Envs (C1080, CRF250, Q23, 25710, CE1176, CH119) were tested for their sensitivity to neutralization by the human V2-apex precursor CH01_RUA. The dashed line indicates 50% reduction in virus infectivity and the corresponding 50% inhibitory concentrations (IC_{50} values in µg/ml) are listed on the right. HIV-1 pseudoviruses sensitive to CH01_RUA are colored in blue, while resistant strains are shown in green. CAM13K (red) and MLV (black) pseudotypes are shown for control.
