# Supplementary material for: A Germline-Targeting Chimpanzee SIV Envelope Glycoprotein Elicits a New Class of V2-Apex Directed Cross-Neutralizing Antibodies
Source: mBio. 2023 Jan 11;14(1):e03370-22. doi: 10.1128/mbio.03370-22 (PMC9973348; doi:10.1128/mbio.03370-22)
Supplement: FIG S3 [file mbio.03370-22-s0003.pdf]

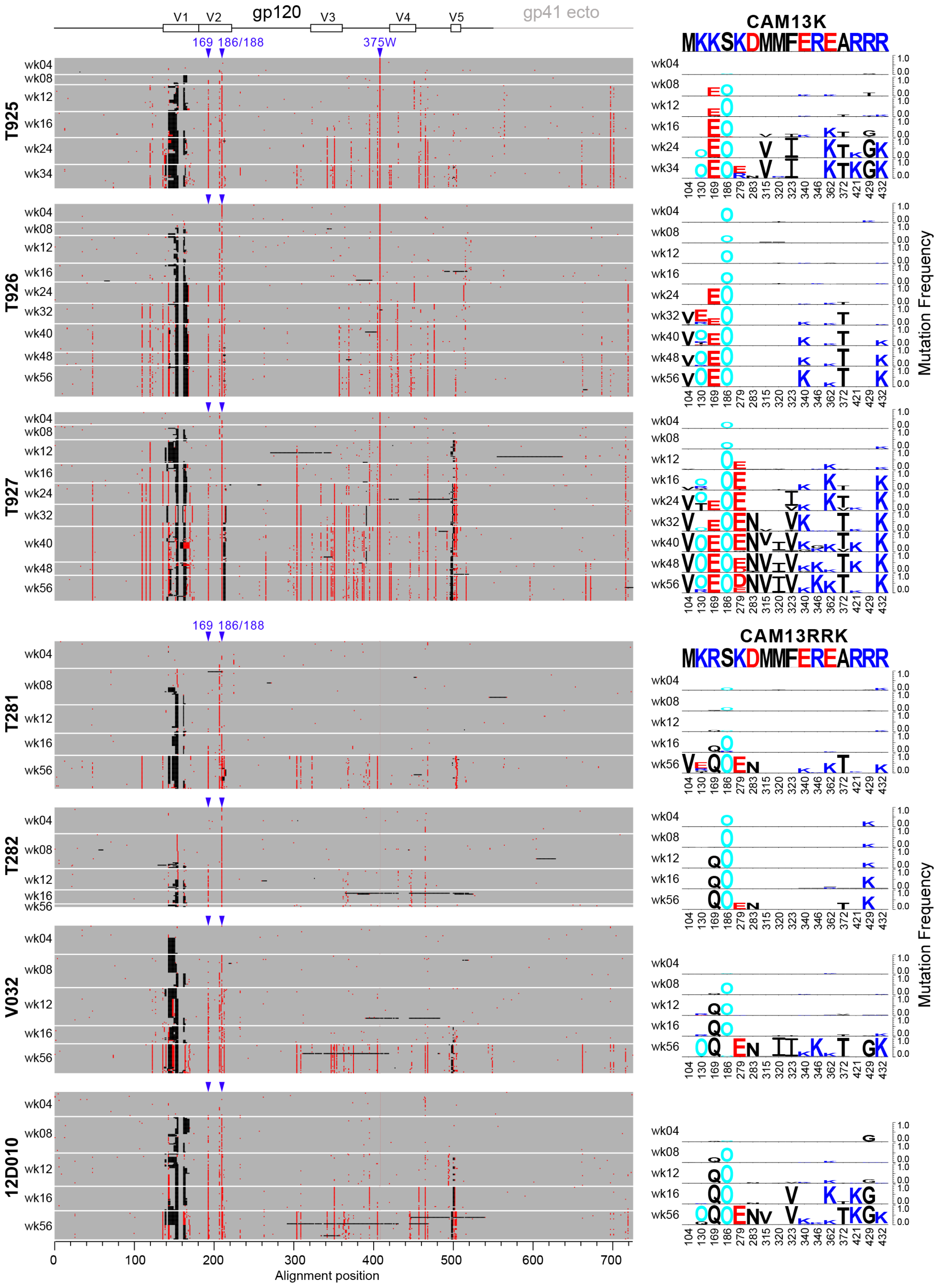

gp120
gp41 ecto
V1
V2
V3
V4
V5
169
186/188
375W
T925
T926
T927
T281
T282
V032
12D010
wk04
wk08
wk12
wk16
wk24
wk32
wk34
wk40
wk48
wk56
Alignment position
CAM13K
MKKSKDMMFEREARRR
CAM13RRK
MKRSKDMMFEREARRR
Mutation Frequency
104 130 169 186 279 283 315 320 323 340 346 362 372 421 429 432

FIG S3 Env sequence evolution in SCIV-infected rhesus macaques. A Highlighter plot of longitudinal Env amino acid sequences obtained by single genome sequencing of plasma viral RNA is shown on the left for each infected RM. Sequences are grouped for each time point (SCIV.CAM13K infected animals are shown on the top, SCIV.CAM13RRK infected animals are shown on the bottom). Each row within a time block represents a single sequence, depicted as a string of horizontal pixels. Thus, each pixel represents a single amino acid in the alignment, which is colored grey if it matches the infecting virus, red if it represents a mutated residue, and black if it is inserted or deleted relative to the infecting virus. Sequences are shown up to HXB2 site 683 (gp41 ectodomain), with a schematic map depicted at the top and an alignment position scale depicted at the bottom. A single alignment was generated from sequences of all RMs and all time points, so that perfectly vertical "stripes" indicate the same HXB2 site in different Envs. One of these stripes represents the outgrowth of the 375W variant from the initial inoculum of six isogenic SCIV.CAM13K mutants (indicated on the top). Positions 169 and 186/188, which are mutated in all animals, are also highlighted. The right panels show mutation frequencies for each time point and RM at sites identified by LASSIE to be selected in at least two rhesus macaques (sites beyond HXB2 position 683 are not shown). Mutations are depicted as logos, where the height of the letter is proportional to its frequency in the Env sequences from the corresponding time point (amino acids of the infecting virus are blanked out). Red, dark blue, and black indicate acidic, basic, and neutral residues, respectively. Cyan colored "O" indicates asparagine (N) embedded in an N-linked glycosylation motif (Asn-X-Ser or Asn-X-Thr, where X can be any amino acid except Pro). Numbers at the bottom indicate residue positions (HXB2 numbering).
