# Supplementary material for: A Germline-Targeting Chimpanzee SIV Envelope Glycoprotein Elicits a New Class of V2-Apex Directed Cross-Neutralizing Antibodies
Source: mBio. 2023 Jan 11;14(1):e03370-22. doi: 10.1128/mbio.03370-22 (PMC9973348; doi:10.1128/mbio.03370-22)
Supplement: FIG S4 [file mbio.03370-22-s0004.pdf]

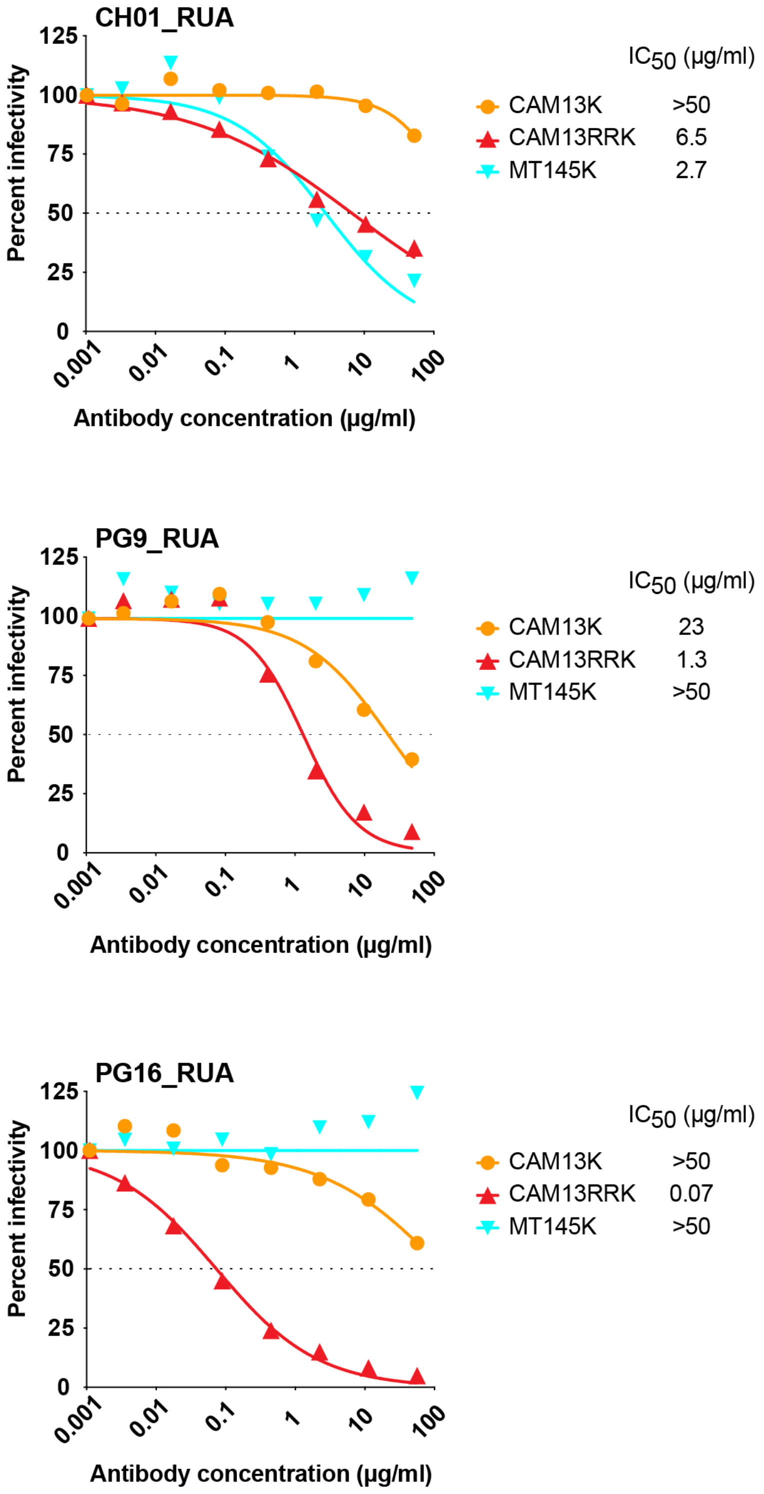

FIG S4 Sensitivity of CAM13RRK to neutralization by V2-apex bNAb precursors. Neutralization curves are shown for human V2-apex bNAb precursors (indicated on top) against CAM13K (CAM13_Q171K), CAM13RRK (CAM13_Q171K_K169R_K170R) and MT145K

(MT145_Q171K) Env bearing pseudoviruses, all of which encode the wildtype methionine amino acid at position 375. The dashed line indicates 50% reduction in virus infectivity and the corresponding 50% inhibitory concentrations (IC_{50} values in μg/ml) are listed on the right.
