# Supplementary material for: A Germline-Targeting Chimpanzee SIV Envelope Glycoprotein Elicits a New Class of V2-Apex Directed Cross-Neutralizing Antibodies
Source: mBio. 2023 Jan 11;14(1):e03370-22. doi: 10.1128/mbio.03370-22 (PMC9973348; doi:10.1128/mbio.03370-22)
Supplement: FIG S5 [file mbio.03370-22-s0005.pdf]

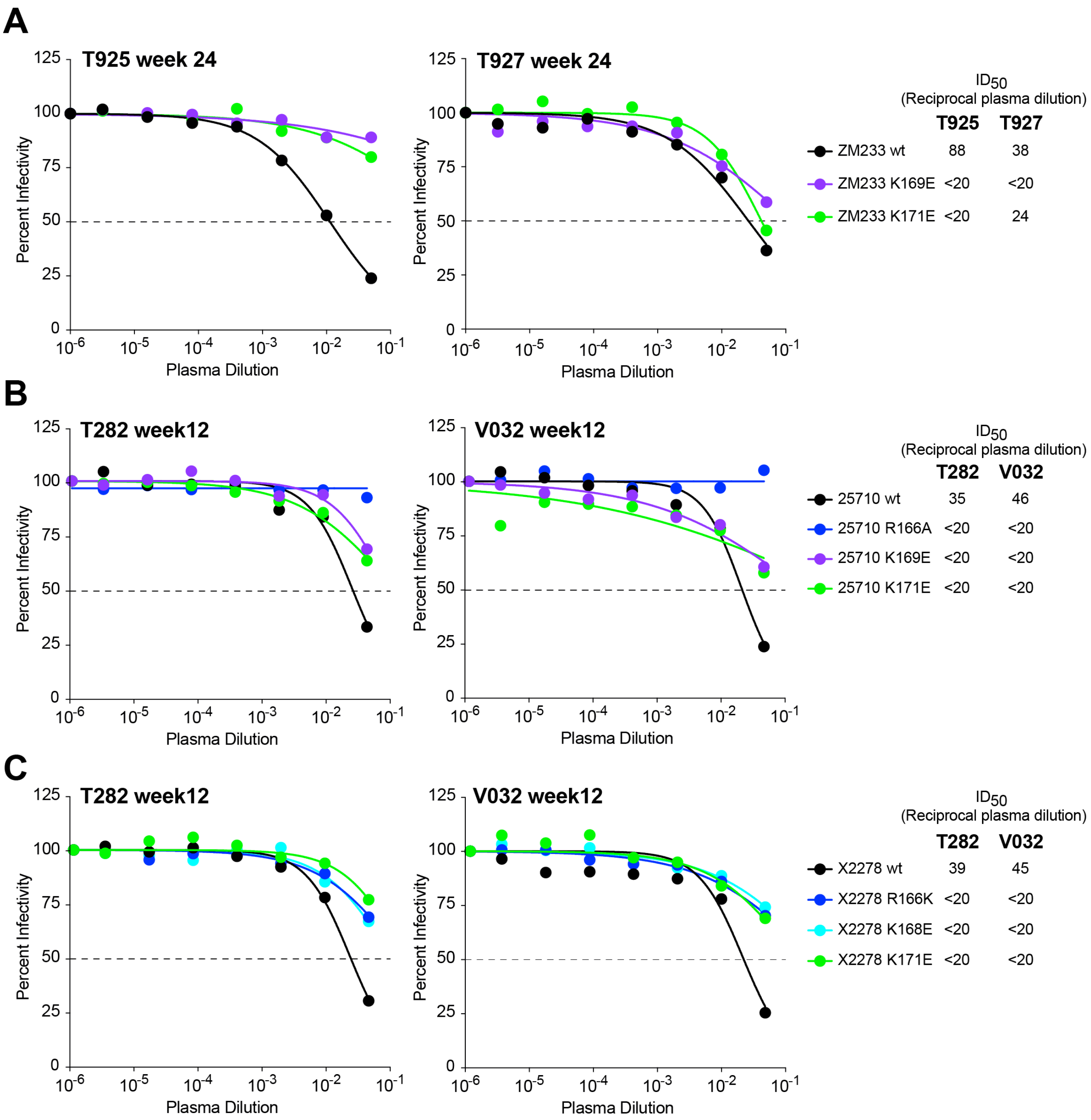

FIG S5 Epitope mapping of plasma neutralizing antibodies in SCIV-infected RMs. Neutralization curves are shown for early plasma samples (weeks 12 and 24) from four RMs (indicated on the top) that developed low-titer heterologous neutralization breadth (also see Fig. 3). Site-directed mutants of ZM233, 25710 and X2278 Envs (indicated on the right) show that

changes at residues 166, 169 and/or 171 reduce plasma neutralization, indicating that the low-titer heterologous plasma breadth maps to the V2-apex. Corresponding ID_{50} values are shown on the right.
