# Supplementary material for: A Germline-Targeting Chimpanzee SIV Envelope Glycoprotein Elicits a New Class of V2-Apex Directed Cross-Neutralizing Antibodies
Source: mBio. 2023 Jan 11;14(1):e03370-22. doi: 10.1128/mbio.03370-22 (PMC9973348; doi:10.1128/mbio.03370-22)
Supplement: FIG S6 [file mbio.03370-22-s0006.pdf]

A

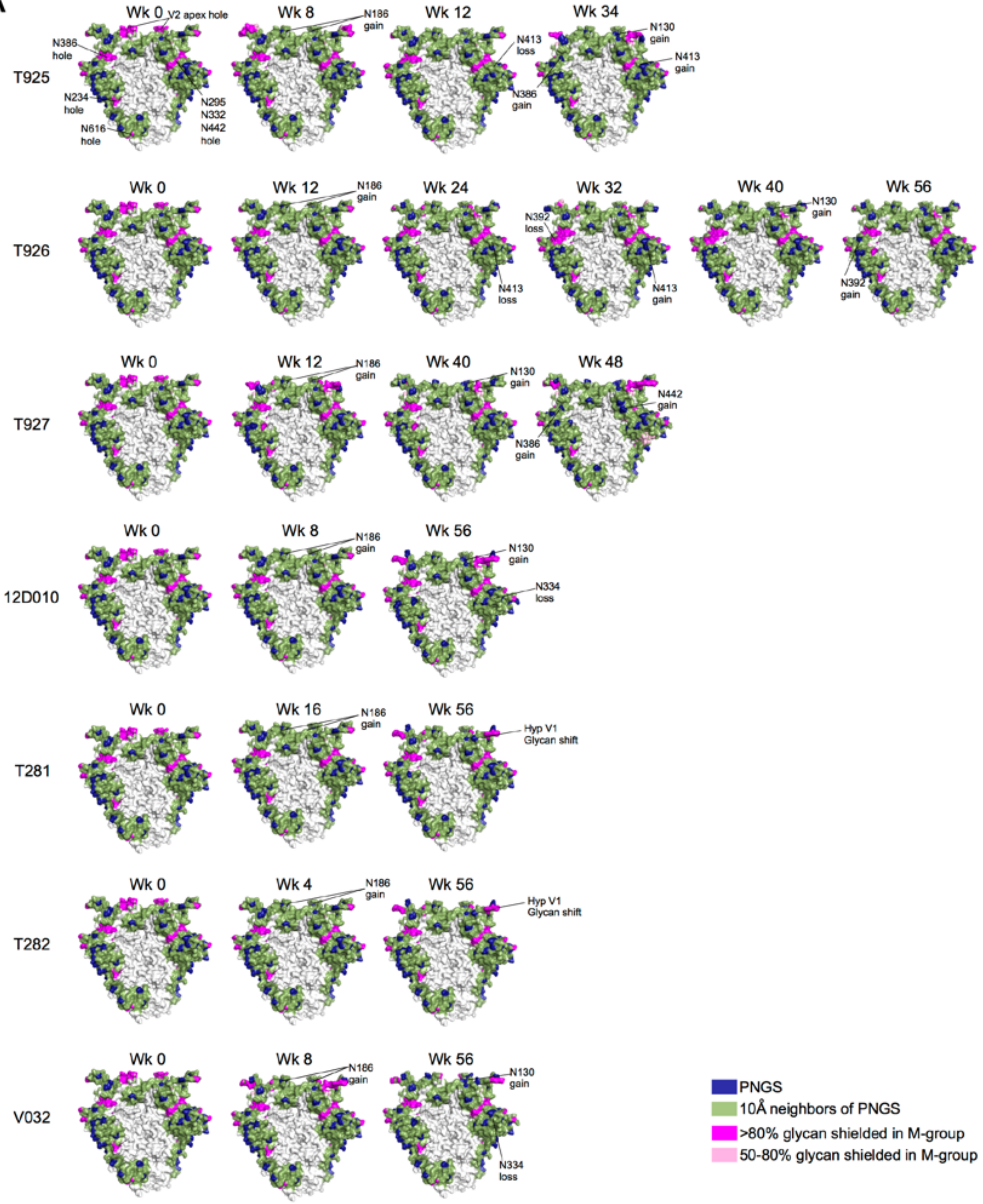

T925
Wk 0
V2 apex hole
N386 hole
N234 hole
N616 hole
N295 N332 N442 hole
Wk 8
N186 gain
Wk 12
N413 loss
Wk 34
N130 gain
N413 gain
N386 gain
T926
Wk 0
Wk 12
N186 gain
Wk 24
N413 loss
Wk 32
N392 loss
N413 gain
Wk 40
N130 gain
Wk 56
N392 gain
T927
Wk 0
Wk 12
N186 gain
Wk 40
N130 gain
N386 gain
Wk 48
N442 gain
12D010
Wk 0
Wk 8
N186 gain
Wk 56
N130 gain
N334 loss
T281
Wk 0
Wk 16
N186 gain
Wk 56
Hyp V1 Glycan shift
T282
Wk 0
Wk 4
N186 gain
Wk 56
Hyp V1 Glycan shift
V032
Wk 0
Wk 8
N186 gain
Wk 56
N130 gain
N334 loss
PNGS
10Å neighbors of PNGS
>80% glycan shielded in M-group
50-80% glycan shielded in M-group

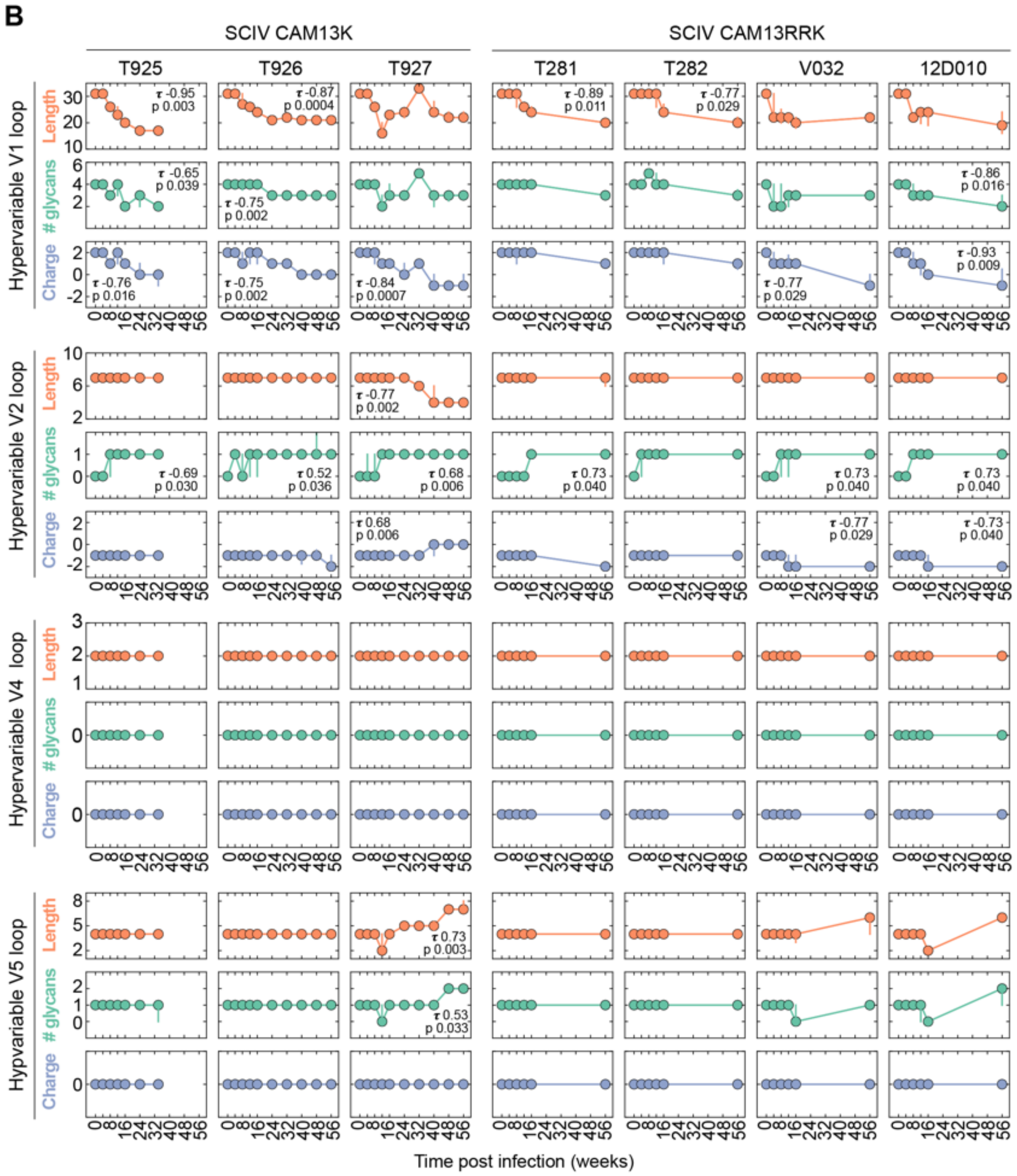

FIG S6 Glycan shield and hypervariable loop evolution in SCIV-infected RMs. (A) Glycan shield evolution. The evolution of the glycan shield is shown for each RM (indicated on the left),

starting with the Env of the transmitted virus and followed by glycan shield calculations of subsequent time points using time-point consensus glycans. The glycan shield is mapped on to a trimeric Env structure oriented with the V2 apex at the top and the viral membrane at the bottom (not shown). Blue regions indicate the location of potential N-linked glycosylation sites, green regions indicate predicted glycan shield coverage, and pink or magenta indicate rare "glycan holes" that are shielded in more than 50% or 80% M-group Envs, respectively. Glycan shield calculations were performed using the Glycan Shield Mapping webtool on the Los Alamos HIV Database (https://www.hiv.lanl.gov/content/sequence/GLYSHIELDMAP/glyshieldmap.html) with consensus glycans (50% or higher frequency for the time point) and hypervariable loop lengths chosen for longitudinal Envs as previously described (56). Glycan gains, losses and shifts are indicated. (B) Env hypervariable loop evolution. The longitudinal evolution of hypervariable loops was examined using alignment-free characteristics, including length, number of glycans and net charge, so as to not bias results by insertion and deletion-related alignment ambiguities. Each RM is depicted as a column and hypervariable loop characteristics are depicted in rows. Individual plots show the longitudinal evolution of the indicated hypervariable loop characteristic (on the left), with time since infection (in weeks) indicated on the bottom. Points show the median of the characteristic calculated using Env sequences from the respective time point, with vertical bars spanning the inter-quartile range (25th to 75th percentiles). Hypervariable loop net charge is measured in amino acid charge units, where His, Lys and Arg have +1 charge, and Asp and Glu have -1 charge. To measure statistical significance of longitudinal trends, the median values for each hypervariable loop characteristic from each time point and RM were subjected to a non-parametric Kendall-Tau test for associations with time post infection; for statistically significant ($p < 0.05$) associations, the Kendall Tau (τ) coefficient and p-value are indicated in the plot. Hypervariable loop positions in HXB2 are: 132-152 for hypervariable V1, 185-190 for hypervariable V2, 396-410 for hypervariable V4 and 460-465 for hypervariable V5. Note that V3 does not have a hypervariable region. All calculations were performed using the Variable Region

Characteristics tool (https://www.hiv.lanl.gov/content/sequence/VAR_REG_CHAR/index.html) available at the Los Alamos HIV Database.
