# Supplementary material for: A Germline-Targeting Chimpanzee SIV Envelope Glycoprotein Elicits a New Class of V2-Apex Directed Cross-Neutralizing Antibodies
Source: mBio. 2023 Jan 11;14(1):e03370-22. doi: 10.1128/mbio.03370-22 (PMC9973348; doi:10.1128/mbio.03370-22)
Supplement: FIG S7 [file mbio.03370-22-s0007.pdf]

A

mAb	MLV	MW965.26	TH023.6	TRO11	25710	CNE8	X2278	BJOX010000	X1632	Ce1176	246F3	CH119	Ce0217	CNE55	Q23	CRF250	WITO	ZM233	CAP256SU	CH505	CH848	BG505 N332*	MT145K
		Tier 1A		Global Neutralization Panel											SHIV Panel								SIVcpz
**T927 lineage 1**																							
w32 P2D12	>250	>250	6.6	>250	>250	>250	>250	>250	>250	>250	>250	>250	>250	>250	>250	>250	>250	>250	>250	>250	>250	>250	>250
**T927 lineage 2**																							
w24 P1D08	>250	0.04	>250	>250	>250	>250	>250	>250	>250	>250	>250	>250	>250	>250	>250	>250	>250	194	>250	>250	>250	>250	>250
w24 P2D06	>250	0.02	>250	>250	>250	>250	>250	>250	>250	>250	>250	>250	>250	>250	>250	>250	>250	119	>250	>250	>250	>250	>250
w24 P1D09	>250	0.02	>250	>250	>250	>250	>250	>250	>250	>250	>250	>250	>250	>250	>250	>250	>250	119	>250	>250	>250	>250	>250
**T927 lineage 3**																							
w62 P1C05	>250	0.05	0.80	>250	59	>250	>250	>250	>250	>250	>250	>250	>250	>250	>250	>250	>250	82	>250	>250	>250	>250	>250
w62 P1C01	>250	0.04	6.9	>250	20.2	>250	>250	>250	>250	>250	>250	>250	>250	>250	>250	>250	>250	40.5	>250	>250	>250	>250	>250
**T925 lineage 2**																							
w24 P1G03	>250				>250										>250			>250					
w24 P1E11	>250				>250										>250			>250					
w24 P1F01	>250				>250										>250			>250					
w24 P2C12	>250				>250										>250			>250					
w24 P1F12	>250				>250										>250			>250					

IC_{50} 250-50μg/ml 49.9-10.0μg/ml 9.9-1.0μg/ml 0.99-0.01μg/ml

B

mAb	CAM13K	CAM13K-K130N	CAM13K-N156K	CAM13K-N160K	CAM13K-R166S	CAM13K-K169E	CAM13K-K171Q	CAM13K-S186N
**T927 lineage 1**								
w32 P2D12	188	140	2.3	141	87	162	>250	233
**T927 lineage 2**								
w24 P1D08	11.5	56	0.05	0.36	>250	>250	>250	40.4
w24 P2D06	9.2	34.6	0.03	0.12	>250	>250	174	30.1
w24 P1D09	10.1	12.4	0.01	0.57	>250	>250	78	27.4
**T927 lineage 3**								
w62 P1C05	>250	>250	9.5	>250	>250	>250	>250	>250
w62 P1C01	>250	>250	11.4	>250	>250	>250	>250	>250
**T927 lineage 4**								
w62 P1B01	>250	>250	6.9	>250	>250	>250	>250	>250
w62 P1B10	>250	>250	17.1	>250	n.d.	>250	>250	>250
w62 P3E08	>250	>250	7.2	170	165	169	>250	>250
w62 P1H10	>250	>250	19.0	>250	>250	>250	>250	>250
w62 P1F05	>250	>250	6.1	>250	>250	>250	>250	>250
w62 P3C02	215	142	1.7	141	110	231	233	198
w62 P1C07	>250	>250	5.0	>250	>250	>250	>250	>250
w62 P3A11	>250	>250	5.8	>250	>250	>250	>250	>250
**T927 lineage 5**								
w24 P2B12	>250	>250	50.0	>250	>250	>250	>250	>250
w24 P1C02	>250	>250	41.2	>250	>250	>250	>250	>250
w24 P1D03	100	>250	5.8	>250	>250	>250	>250	>250
w24 P1A11	13.6	100	1.4	>250	>250	>250	>250	48.0
w62 P1B05	157	>250	1.3	>250	>250	>250	>250	>250
w62 P3G11	161	>250	1.2	>250	>250	>250	>250	>250
**T925 lineage 1**								
w24 P1E09	1.2	3.3	0.04	>250	>250	>250	>250	6.6
w24 P1D09	1.1	6.8	0.09	>250	>250	>250	>250	4.6
w24 P2B11	2.3	14.0	0.10	102	>250	>250	>250	5.9
w24 P1A12	0.6	4.4	0.05	219	>250	>250	>250	3.7
w24 P1C08	6.6	48.3	0.46	>250	>250	>250	>250	45.4
**T925 lineage 2**								
w24 P1G03	>250							
w24 P1E11	>250							
w24 P1F01	>250							
w24 P2C12	>250							
w24 P1F12	>250							

mAb	ZM233	ZM233-N160K	ZM233-R166S	ZM233-K168E	ZM233-K169E	ZM233-K171E	MW965.26	MW965.26-N160K	MW965.26-R166A	MW965.26-K168E	MW965.26-K169E	MW965.26-Q171K
**T927 lineage 5**												
w24 P2B12	14.0	48.0	>250	>250	>250	>250	1.4	>10	>10	>10	>10	0.91
w24 P1C02	68	61	>250	>250	>250	>250	3.5	>10	>10	>10	>10	1.0
w24 P1D03	10.1	21.3	>50	>50	>50	>50	0.76	6.8	>10	>10	>10	0.22
w24 P1A11	0.52	1.2	>50	>50	>50	>50	0.09	0.73	>10	>10	>10	0.01
w62 P1B05	0.62	5.3	>50	>50	46.0	>50	0.01	0.30	>10	>10	0.01	0.003
w62 P3G11	2.0	5.3	>50	>50	>50	>50	0.14	2.5	>10	>10	0.37	0.02
**T925 lineage 1**												
w24 P1E09	0.78	9.5	17.8	>50	>50	>50	4.0	>10	>10	>10	>10	0.007
w24 P1D09	5.0	2.5	>50	>50	>50	>50	9.0	>10	>10	>10	>10	0.009
w24 P2B11	0.77	18.7	17.0	>50	>50	>50	4.9	>10	>10	>10	>10	0.02
w24 P1A12	0.28	13.0	13.0	>50	>50	>50	22.9*	>10	>10	>10	>10	0.006
w24 P1C08	2.0	9.7	>50	>50	>50	>50	>10	>10	>10	>10	>10	0.05
**T925 lineage 2**												
w24 P1G03	>250											
w24 P1E11	>250											
w24 P1F01	>250											
w24 P2C12	>250											
w24 P1F12	>250											

IC_{50} 250-50μg/ml 49.9-10.0μg/ml 9.9-1.0μg/ml 0.99-0.01μg/ml <0.01μg/ml

FIG S7 Neutralization properties of antibody lineages isolated from SCIV-infected RMs. (A) Expanded antibody lineages isolated from SCIV.CAM13K infected RMs with no or very limited tier 2 neutralization breadth. 50% inhibitory concentrations (IC_{50}) are shown (μg/ml) for representative members of T927 lineages 1-3 and T925 lineage 2 (listed on the left) against

pseudovirus panels carrying HIV-1 and SIVcpz Envs as well as replicating SHIVs (indicated on top). The highest antibody concentration used was 250 μg/ml (coloring indicates the relative neutralization potency). (B) Epitope mapping of SCIV-induced cross-neutralizing antibodies. Representative members of all isolated antibody lineages (listed on the left) were tested against panels of CAM13K, ZM233 and/or MW965.26 mutants (indicated on top). Given the patterns of early sequence evolution, we mutagenized residues at or near the V2-apex, with an emphasis on key N-linked glycosylation sites and C strand residues. 50% inhibitory concentrations (IC_{50}) are shown (μg/ml). An asterisk denotes an estimated IC_{50} value (as determined by the Prism software). Only the two most potent and cross-reactive lineages were tested against heterologous ZM233 and MW965.26 mutants. All viruses represent pseudotypes generated by transfection of 293T cells. The highest antibody concentration used was 250 μg/ml (colors indicate the relative neutralization potency).
