# Supplementary material for: A Germline-Targeting Chimpanzee SIV Envelope Glycoprotein Elicits a New Class of V2-Apex Directed Cross-Neutralizing Antibodies
Source: mBio. 2023 Jan 11;14(1):e03370-22. doi: 10.1128/mbio.03370-22 (PMC9973348; doi:10.1128/mbio.03370-22)
Supplement: FIG S8 [file mbio.03370-22-s0008.pdf]

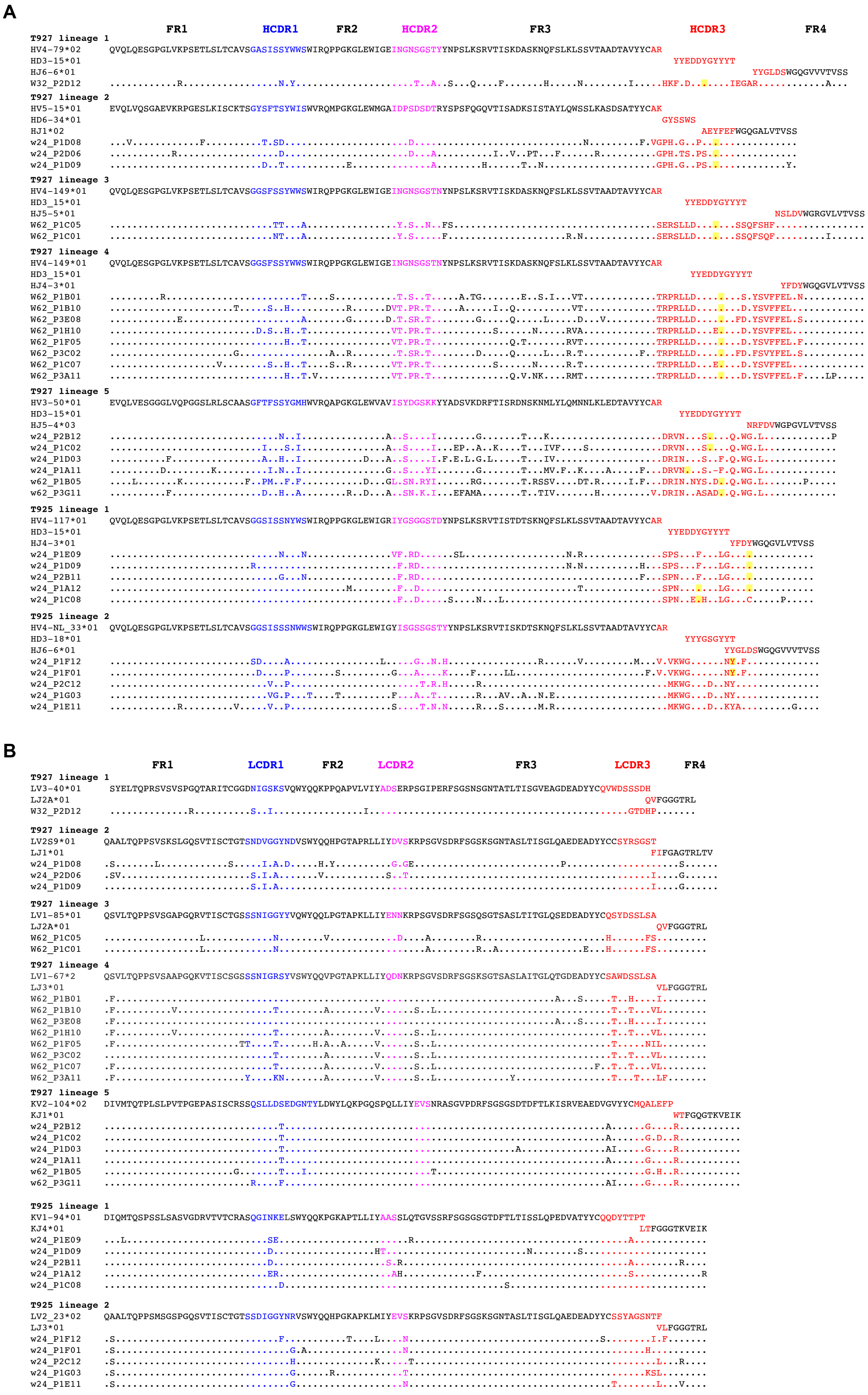
A
FR1 HCDR1 FR2 HCDR2 FR3 HCDR3 FR4
T927 lineage 1
HV4-79*02 QVQLQESGPGLVKPSETLSLTCAVSGASISSYWWSWIRQPPGKGLEWIGEINGNSGSTYYNPSLKSRVTISKDASKNQFSLKLSSVTAADTAVYYCAR
HD3-15*01 YYEDDYGYYYT
HJ6-6*01 YYGLDSWGQGVVVTVSS
W32_P2D12
T927 lineage 2
HV5-15*01 EVQLVQSGAEVKRPGESLKISCKTSGYSFTSYWISWVRQMPGKGLEWMGAIDPSDSDTRYSPSFQGQVTISADKSISTAYLQWSSLKASDSATYYCAK
HD6-34*01 GYSSWS
HJ1*02 AEYFEFWGQGALVTVSS
w24_P1D08
w24_P2D06
w24_P1D09
T927 lineage 3
HV4-149*01 QVQLQESGPGLVKPSETLSLTCAVSGGSFSSYWWSWIRQPPGKGLEWIGEINGNSGSTNYNPSLKSRVTISKDASKNQFSLKLSSVTAADTAVYYCAR
HD3_15*01 YYEDDYGYYYT
HJ5-5*01 NSLDVWGRGVLVTVSS
W62_P1C05
W62_P1C01
T927 lineage 4
HV4-149*01 QVQLQESGPGLVKPSETLSLTCAVSGGSFSSYWWSWIRQPPGKGLEWIGEINGNSGSTNYNPSLKSRVTISKDASKNQFSLKLSSVTAADTAVYYCAR
HD3_15*01 YYEDDYGYYYT
HJ4-3*01 YFDYWGQGVLVTVSS
W62_P1B01
W62_P1B10
W62_P3E08
W62_P1H10
W62_P1F05
W62_P3C02
W62_P1C07
W62_P3A11
T927 lineage 5
HV3-50*01 EVQLVESGGGLVQPGGSLRLSCAASGFTFSSYGMHWVRQAPGKGLEWVAVISYDGSKKYYADSVKDRFTISRDNSKNMLYLQMNNLKLEDTAVYYCAR
HD3-15*01 YYEDDYGYYYT
HJ5-4*03 NRFDVWGPGVLVTVSS
w24_P2B12
w24_P1C02
w24_P1D03
w24_P1A11
w62_P1B05
w62_P3G11
T925 lineage 1
HV4-117*01 QVQLQESGPGLVKPSETLSLTCAVSGGSISSNYWSWIRQPPGKGLEWIGRIYGSGGSTDYNPSLKSRVTISTDTSKNQFSLKLSSVTAADTAVYYCAR
HD3-15*01 YYEDDYGYYYT
HJ4-3*01 YFDYWGQGVLVTVSS
w24_P1E09
w24_P1D09
w24_P2B11
w24_P1A12
w24_P1C08
T925 lineage 2
HV4-NL_33*01 QVQLQESGPGLVKPSETLSLTCAVSGGSISSSNWWSWIRQPPGKGLEWIGYISGSSGSTYYNPSLKSRVTISKDTSKNQFSLKLSSVTAADTAVYYCAR
HD3-18*01 YYYGSGYYT
HJ6-6*01 YYGLDSWGQGVVVTVSS
w24_P1F12
w24_P1F01
w24_P2C12
w24_P1G03
w24_P1E11
B
FR1 LCDR1 FR2 LCDR2 FR3 LCDR3 FR4
T927 lineage 1
LV3-40*01 SYELTQPRSVSVSPGQTARITCGGDNIGSKSVQWYQQKPPQAPVLVIYADSERPSGIPERFSGSNSGNTATLTISGVEAGDEADYYCQVWDSSSDH
LJ2A*01 QVFGGGTRL
W32_P2D12
T927 lineage 2
LV2S9*01 QAALTQPPSVSKSLGQSVTISCTGTSNDVGGYNDVSWYQQHPGTAPRLLIYDVSKRPSGVSDRFSGSKSGNTASLTISGLQAEDEADYYCCSYRSGST
LJ1*01 FIFGAGTRLTV
w24_P1D08
w24_P2D06
w24_P1D09
T927 lineage 3
LV1-85*01 QSVLTQPPSVSGAPGQRVTISCTGSSSNIGGYYVQWYQQLPGTAPKLLIYENNKRPSGVSDRFSGSQSGTSASLTITGLQSEDEADYYCQSYDSSLSA
LJ2A*01 QVFGGGTRL
W62_P1C05
W62_P1C01
T927 lineage 4
LV1-67*2 QSVLTQPPSVSAAPGQKVTISCSGSSSNIGRSYVSWYQQVPGTAPKLLIYQDNKRPSGVSDRFSGSKSGTSASLAITGLQTGDEADYYCSAWDSSLSA
LJ3*01 VLFGGGTRL
W62_P1B01
W62_P1B10
W62_P3E08
W62_P1H10
W62_P1F05
W62_P3C02
W62_P1C07
W62_P3A11
T927 lineage 5
KV2-104*02 DIVMTQTPLSLPVTPGEPASISCRSSQSLLDSEDGNTYLDWYLQKPGQSPQLLIYEVSNRASGVPDRFSGSGSDTDFTLKISRVEAEDVGVYYCMQALEFP
KJ1*01 WTFGQGTKVEIK
w24_P2B12
w24_P1C02
w24_P1D03
w24_P1A11
w62_P1B05
w62_P3G11
T925 lineage 1
KV1-94*01 DIQMTQSPSSLSASVGDRVTVTCRASQGINKELSWYQQKPGKAPTLLIYAASSLQTGVSSRFSGSGSGTDFTLTISSLQPEDVATYYCQQDYTTPT
KJ4*01 LTFGGGTKVEIK
w24_P1E09
w24_P1D09
w24_P2B11
w24_P1A12
w24_P1C08
T925 lineage 2
LV2_23*02 QAALTQPPSMSGSPGQSVTISCTGTSSDIGGYNRVSWYQQHPGKAPKLMIYEVSKRPSGVSDRFSGSKSGNTASLTISGLQAEDEADYYCSSYAGSNTF
LJ3*01 VLFGGGTRL
w24_P1F12
w24_P1F01
w24_P2C12
w24_P1G03
w24_P1E11

FIG. S8 Heavy and light chain variable region sequences of antibody lineages isolated from SCIV-infected RMs. (A, B) Heavy (H, panel A) and light (lambda [L] and kappa [K], panel B) chain variable region sequences of mature lineage members isolated from RMs T927 and T925 (listed on the left) are aligned to the deduced amino acid sequences of their respective germline variable (V), diversity (D), and joining (J) genes (indicated on the top). Framework (FR) and complementary determining regions (CDR) are color coded, with HCDR1 and LCDR1, HCDR2 and LCDR2, and HCDR3 and LCDR3 highlighted in blue, pink and red, respectively. Predicted sulfated tyrosines in the HCDR3 region (using the GPS-TSP 1.0 software at a high threshold setting) are highlighted in yellow (98). Expanded lineages (listed on the left) were identified using SONAR's automated "unseeded" lineage assignment tool, followed by manual inspection of gene assignments, HCDR3 regions, and non-templated nucleotides to confirm membership (Genbank accession numbers are listed in Table S2E at https://doi.org/10.6084/m9.figshare.21644675).
