# Supplementary material for: A Germline-Targeting Chimpanzee SIV Envelope Glycoprotein Elicits a New Class of V2-Apex Directed Cross-Neutralizing Antibodies
Source: mBio. 2023 Jan 11;14(1):e03370-22. doi: 10.1128/mbio.03370-22 (PMC9973348; doi:10.1128/mbio.03370-22)
Supplement: FIG S9 [file mbio.03370-22-s0009.pdf]

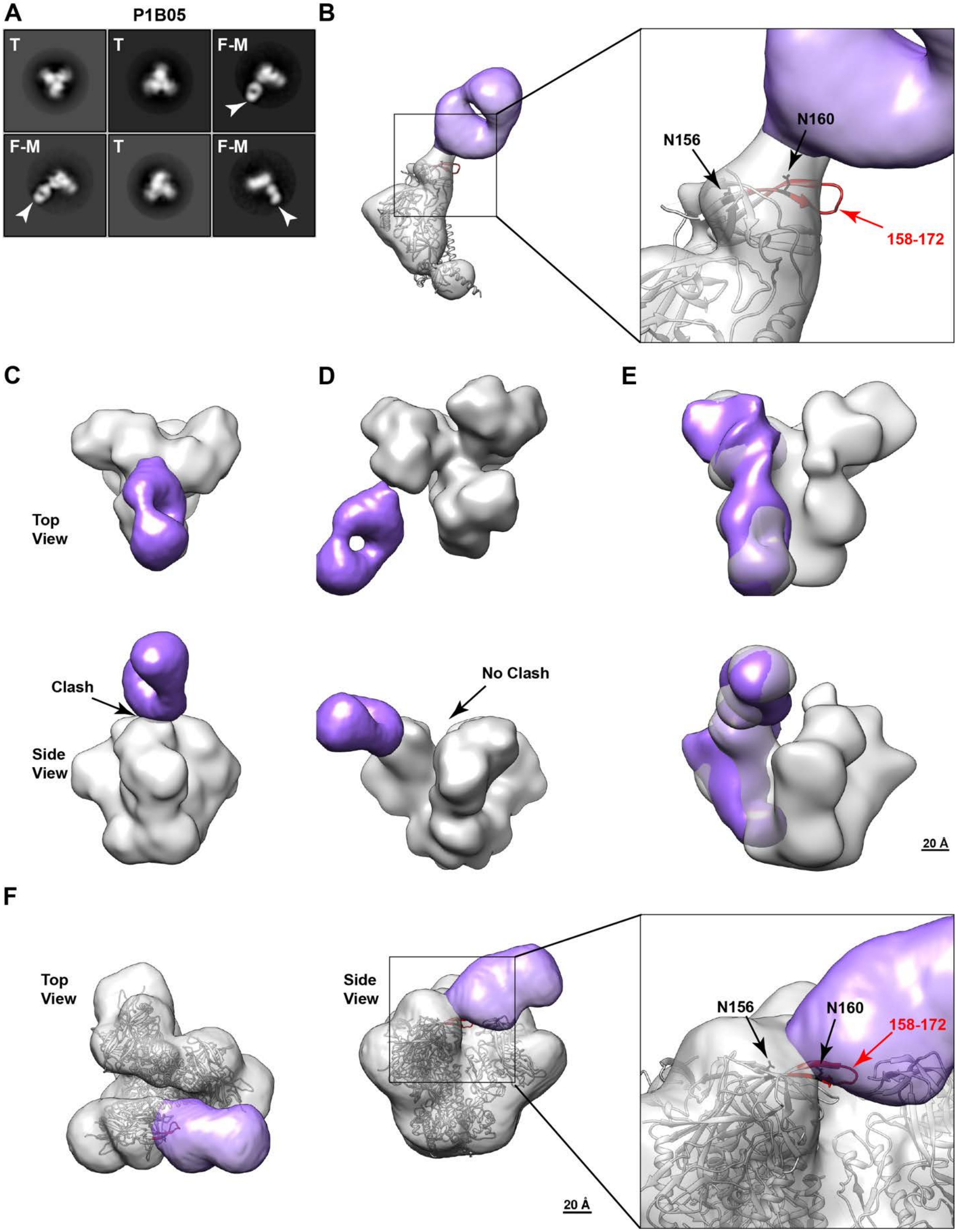
A
P1B05
T
T
F-M
F-M
T
F-M
B
N156
N160
158-172
C
Top View
Side View
Clash
D
No Clash
E
20 Å
F
Top View
Side View
N156
N160
158-172
20 Å

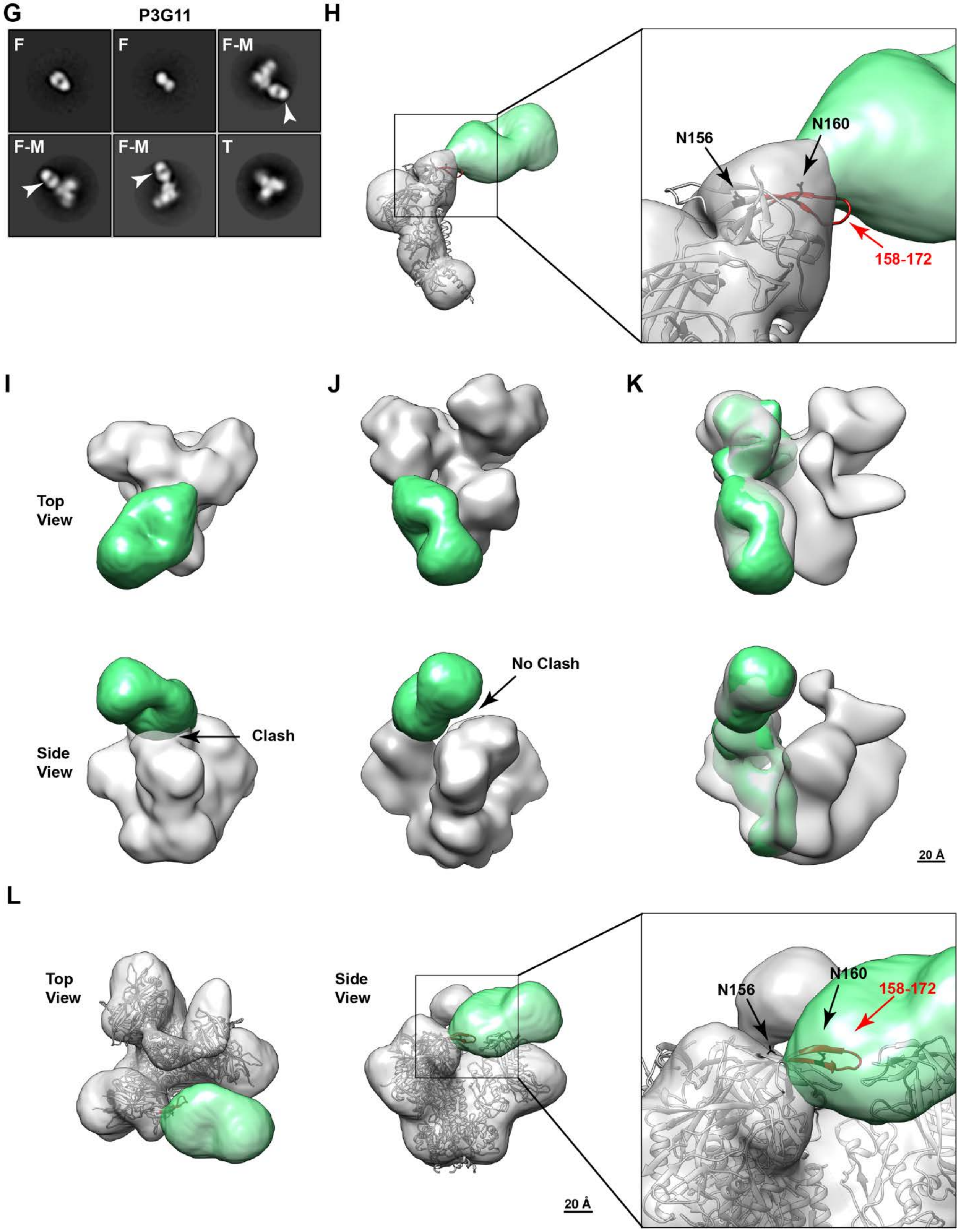

G
P3G11
F
F
F-M
F-M
F-M
T
H
N156
N160
158-172
I
J
K
Top View
Side View
Clash
No Clash
20 Å
L
Top View
Side View
N156
N160
158-172
20 Å

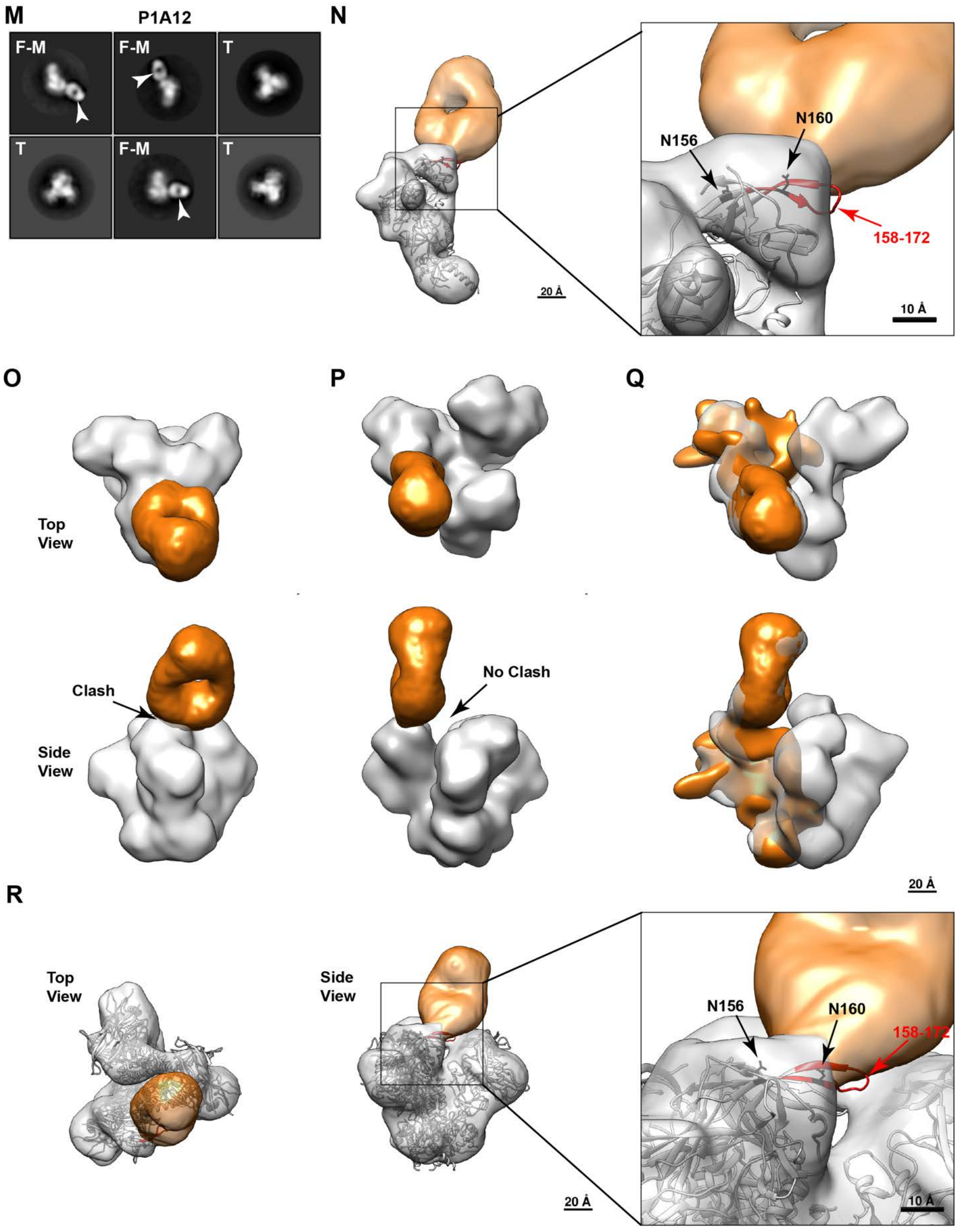
M
P1A12
F-M
F-M
T
T
F-M
T
N
N156
N160
158-172
20 Å
10 Å
O
P
Q
Top View
Side View
Clash
No Clash
20 Å
R
Top View
Side View
N156
N160
158-172
20 Å
10 Å

FIG S9 Negative stain EM analysis of SCIV-induced cross-neutralizing antibodies. (panels A, G and H) 2D class averages showing intact SOSIP trimers without bound Fabs (T), Fabs (F) and Fab-monomers (F-M), but no Fab-trimer complexes. The antibody from which the Fabs are derived are indicated on top. Fabs in Fab-monomer complexes are indicated by an arrow. (panels B, H and N) 3D reconstruction of a Fab-monomers fit the model of a single Env protomer (PDB 5FYL), with the Env domain in gray and the Fab in color (P1B05, purple; P3G11, green; P1A1, gold). A higher magnification inset identifies residues 158-172 in the V2 loop (highlighted in red) as the likely epitope (the positions of glycans at positions 156 and 160 are shown). (panels C, I and O) Modeling of Fab binding onto a closed, prefusion trimer. Both top and side views are shown, revealing clashes (arrows) with adjacent protomers. (panels D, J and P) Modeling of Fab binding onto an occluded-open trimer. Both top and side views are shown, identifying no clashes with adjacent protomers. (panels E, K and Q) Overlay of Fab-monomer (colored) and Fab-trimer structures. The Fab-monomer fits well within the density of the corresponding trimer, thus validating the utility of Fab-monomer complexes to infer putative epitopes and approximate angles of approach. (panels F, L and R) Fab-trimer structures identify the same putative V2 epitope (residues 158-172; highlighted in red) as the corresponding Fab-monomer structures shown in panels B, H and N, respectively.
